# Developing a broad-range promoter set for metabolic engineering in the thermotolerant yeast *Kluyveromyces marxianus*

**DOI:** 10.1016/j.mec.2020.e00145

**Published:** 2020-09-03

**Authors:** Xuye Lang, Pamela B. Besada-Lombana, Mengwan Li, Nancy A. Da Silva, Ian Wheeldon

**Affiliations:** a Department of Chemical and Environmental Engineering, UC Riverside, United States; b Department of Chemical and Biomolecular Engineering, UC Irvine, United States; c Center for Industrial Biotechnology, UC Riverside, United States

**Keywords:** Thermo-tolerance, Non-conventional microbe, Promoter, Chemical production, *K. marxianus*

## Abstract

*Kluyveromyces marxianus* is an emerging host for metabolic engineering. This thermotolerant yeast is the fastest growing eukaryote, has high flux through the TCA cycle, and can metabolize a broad range of C5, C6, and C12 carbon sources. In comparison to the common host *Saccharomyces cerevisiae*, this non-conventional yeast suffers from a lack of metabolic engineering tools to control gene expression over a wide transcriptional range. To address this issue, we designed a library of 25 native-derived promoters from *K. marxanius* CBS6556 that spans 87-fold transcriptional strength under glucose metabolism. Six promoters from the library were further characterized in both glucose and xylose as well as across various temperatures from 30 to 45 ​°C. The temperature study revealed that in most cases EGFP expression decreased with elevating temperature; however, two promoters, P_*SSA3*_ and P_*ADH1*_, increased expression above 40 ​°C in both xylose and glucose. The six-promoter set was also validated in xylose for triacetic acid lactone (TAL) production. By controlling the expression level of heterologous 2-pyrone synthase (2-PS), the specific TAL titer increased over 8-fold at 37 ​°C. Cultures at 41 ​°C exhibited a similar TAL biosynthesis capability, while at 30 ​°C TAL levels were lower. Taken together, these results advance the metabolic engineering tool set in *K. marxianus* and further develop this new host for chemical biosynthesis.

## Highlights

• Library of 25 *K. marxianus* promoters spans nearly 2 order magnitude of transcriptional strength under glucose metabolism.
• Promoters show similar strength trends with both glucose and xylose metabolism.
• *KmNC1* promoter shows highest strength among reported *K. marxianus* native promoters.
• *KmNC1* promoter increased specific triacetic acid lactone (TAL) titer by 80% compared with previous studies.

## 1 Introduction

Over the past few decades, the US industrial biotechnology sector has been growing at an average rate of ~10% per year and is estimated to be 2-3% of US gross domestic product (GDP) (Carlson, 2016). The global biotechnology market has been growing at a similar rate and is estimated to be upward of $700 billion by 2025 (Kazmierczak et al., 2019). The continued expansion of this industry requires new bioprocessing technologies and new metabolic engineering hosts that can meet industrial needs. For example, microbial hosts with high native stress tolerance can benefit bioprocessing by enabling high temperature fermentations, the production of toxic solvents and compounds, and the potential use of low cost water sources including industrial wastes and sea water high in salts and other minerals (Thorwall et al., 2020).

Due to its native stress tolerance, the yeast *Kluyveromyces marxianus* is a promising eukaryotic host for bioproduction. Various strains of this species are able to grow at 45 ​°C and above and in media with high salt concentration (≥5% NaCl) (Andreu and Del Olmo, 2020; Nonklang et al., 2008; Yuan et al., 2008; Lane and Morrissey, 2010). *K. marxianus* also benefits from the ability to metabolize a range of C5, C6, and C12 sugars and exhibits the fastest growth rate (~0.7 h^−1^) of any known eukaryote (Löbs et al., 2016, 2017; Rodrussamee et al., 2011). Like other non-conventional microbes, there has been considerably less research effort put towards developing well-characterized genetic parts for manipulating gene expression in comparison to the common laboratory host and ethanol producer *Saccharomyces cerevisiae* (Löbs et al., 2017). Despite this general lack of metabolic engineering tools, simple genetic manipulations and media optimization have been used to demonstrate the potential of *K. marxianus* for a variety of bioprocesses including protein production, high temperature ethanol fermentation, the biosynthesis of ethyl acetate (a native metabolite produced in grams per liter quantities in many *K. marxianus* strains) and heterologous products such as the polyketide triacetic acid lactone (TAL) (Cernak et al., 2018; Raimondi et al., 2013; Kushi et al., 2000; Lertwattanasakul et al., 2011; Yarimizu et al., 2015; Madeira and Gombert, 2018; McTaggart et al., 2019). The development of new metabolic engineering tools (*e.g.*, promoters, gene editing systems, gene integration strategies, etc.) is needed to advance the current successes as well as to develop new strains for the high titer production of chemicals, fuels, and biologics using *K. marxianus* as a microbial host.

It has previously been demonstrated that a number of *S. cerevisiae* promoters can function in *K. marxianus*, but expression level is generally low and their ability to operate at elevated temperatures has not yet been established (Lee et al., 2013). We have leveraged this minimal functionality to adapt CRISPR-Cas9 and CRISPR interference (CRISPRi) systems for use in *K. marxianus*. Specifically, plasmid expression of Cas9 or deactivated Cas9 (dCas9) was accomplished using the heterologous *S. cerevisiae TEF1* promoter (Löbs et al., 2017, 2018). In addition to the development of CRISPR-based genome editing and gene regulation tools, recent efforts have identified a set of native constitutive and inducible promoters in *K. marxianus* (Rajkumar et al., 2019). While these new systems greatly enhance our ability to edit the *K. marxianus* genome and tune gene transcription, there is still a need for a set of well-characterized promoters that span a broad range of expression over a wide temperature spectrum and with different carbon sources.

We have expanded the existing promoter set by characterizing a library of 25 putative promoters comprising the region between the start codon to 700 bp upstream the start codon of selected genes in *K. marxianus* CBS6556 *ΔHIS3 ΔURA3* (Lee et al., 2015). The resulting library enables a range of heterologous protein expression of greater than 80-fold. We also determined promoter dynamics through lag, exponential, and stationary phases, promoter strength in glucose and xylose media, and evaluated temperature effects on expression. Finally, we selected a subset of six promoters that retained a wide range of transcriptional strengths in xylose to overexpress 2-pyrone synthase (2-PS) for the synthesis of the polyketide TAL, and compared the strongest promoter with the *S. cerevisiae ADH2* promoter for high level production.

## 2 Materials and methods

*Media and* c*ultivation*. *Escherichia coli* strains XL-1 Blue and TOP10 were cultivated in 5 ​mL of Luria Bertani (LB) medium containing 50–100 ​μg/mL of ampicillin. Overnight cultures of *K. marxianus* CBS6556 *ΔHIS3 ΔURA3* were grown in 2 ​mL of 2% YPD (10 ​g/L yeast extract (BD Difco™), 20 ​g/L of peptone (BD Difco™) and 20 ​g/L of D-glucose (Fisher Scientific) for plasmid transformation. The *HIS3* disruption was created by CRISPR-Cas9 targeting the *HIS* locus in a previously reported CBS6556 *ΔURA3* strain (Table 1). For expression experiments using *K. marxianus* CBS6556 *ΔHIS3 ΔURA3*, strains were grown in 250 ​mL baffled flasks using 25 ​mL of SD-His (20 ​g/L D-glucose (Fisher Scientific), 6.7 ​g/L yeast nitrogen base (BD Difco™), 0.77 ​g/L CSM-His (Sunrise)) or SX-His (20 ​g/L xylose (Alfa Aestar), 5 ​g/L ammonium sulfate (Fisher Scientific), 1.7 ​g/L yeast nitrogen base without amino acids (BD Difco™), and 0.77 ​g/L CSM-His (Sunrise)) at 250 ​rpm and 30, 37, 41 or 45 ​°C. SD stands for synthetic defined media with dextrose and SX denotes synthetic defined media with xylose. For all media, carbon sources were autoclaved separately, and yeast nitrogen base solutions were filtered. For TAL production experiments using the *K. marxianus* industrial stain KM1 *ΔURA3* (Table 1), cells were grown at 37 ​°C in 15 ​× ​125 ​mm borosilicate culture tubes containing 3 ​mL of 1% SCA media (5 ​g/L casamino acids (BD Difco™), 5 ​g/L ammonium sulfate (Fisher Scientific), 1.7 ​g/L yeast nitrogen base without amino acids (BD Difco™), 100 ​mg/L adenine hemisulfate (Sigma)) and 10 ​g/L xylose (1% SXCA) or 9.5 ​g/L lactose (0.95% SLCA) (providing an equimolar amount of carbon).

**Table 1:** Strains and plasmids.

Name	Description	Reference
**Strains**		
*K. marxianus* CBS6556 *ΔHIS3 ΔURA3*	*his3Δura3Δ*	This study
*K.marxianus* KM1 *ΔURA3*	*ura3Δ*	Pecota, Rajgarhia and Da Silva, 2007
*E. coli* TOP10	F^−^ ​*mcr*A Δ(*mrr*-*hsd*RMS-*mcr*BC) ϕ80*lac*ZΔM15 Δ*lac*X74 ​*rec*A1 ​*ara*D139 Δ(*ara-leu*)7697 ​*gal*U ​*gal*K λ^–^ ​*rps*L (Str^R^) ​*end*A1 ​*nup*G	Thermo Fisher
XL-1 blue	*rec*A1 *end*A1 *gyr*A 96 thi-1 *hsd*R17 *sup*E 44 *rel*A1 *lac* [F′ *pro*AB *lac*Iq ZΔM15 Tn10 (Tetr)]	Thermo Fisher
**Plasmids**		
pIW1198	*K.m.* CEN/ARS, P_*GDPSc*_-GFP-T_*CYC1Sc*_, *HIS3* marker	This study
pIW578	*K.m.* CEN/ARS, P_*GDPSc*_-T_*CYC1Sc*_, *HIS3* marker	This study
pXP842-2PS	2μ plasmid, P_*ADH2Sc*_-2-PS-T_*CYC1Sc*_, *URA3* marker	Cardenas and Da Silva (2014)
P_*ADH1*_-EGFP	*K.m.* CEN/ARS, P_*ADH1*_-EGFP-T_*CYC1Sc*_, *HIS3* marker	This study
P_*INU1*_-EGFP	*K.m.* CEN/ARS, P_*INU1*_-EGFP-T_*CYC1Sc*_, *HIS3* marker	This study
P_*RPE1*_-EGFP	*K.m.* CEN/ARS, P_*RPE1*_-EGFP-T_*CYC1Sc*_, *HIS3* marker	This study
P_*PIR1*_-EGFP	*K.m.* CEN/ARS, P_*PIR1*_-EGFP-T_*CYC1Sc*_, *HIS3* marker	This study
P_*POL4*_-EGFP	*K.m.* CEN/ARS, P_*POL4*_-EGFP-T_*CYC1Sc*_, *HIS3* marker	This study
P_*HSP26*_-EGFP	*K.m.* CEN/ARS, P_*HSP26*_-EGFP-T_*CYC1Sc*_, *HIS3* marker	This study
P_*SSA3*_-EGFP	*K.m.* CEN/ARS, P_*SSA3*_-EGFP-T_*CYC1Sc*_, *HIS3* marker	This study
P_*ZWF*_ -EGFP	*K.m.* CEN/ARS, P_*ZWF*_-EGFP-T_*CYC1Sc*_, *HIS3* marker	This study
P_*SCL1*_-EGFP	*K.m.* CEN/ARS, P_*SCL1*_-EGFP-T_*CYC1Sc*_, *HIS3* marker	This study
P_*ALD2*_-EGFP	*K.m.* CEN/ARS, P_*ALD2*_-EGFP-T_*CYC1Sc*_, *HIS3* marker	This study
P_*PST1*_-EGFP	*K.m.* CEN/ARS, P_*PST1*_-EGFP-T_*CYC1Sc*_, *HIS3* marker	This study
P_*GLK1A*_-EGFP	*K.m.* CEN/ARS, P_*GLK1A*_-EGFP-T_*CYC1Sc*_, *HIS3* marker	This study
P_*COX20*_-EGFP	*K.m.* CEN/ARS, P_*COX20*_-EGFP-T_*CYC1Sc*_, *HIS3* marker	This study
P_*SOD1*_-EGFP	*K.m.* CEN/ARS, P_*SOD1*_-EGFP-T_*CYC1Sc*_, *HIS3* marker	This study
P_*GPD1*_-EGFP	*K.m.* CEN/ARS, P_*GPD****1***_-EGFP-T_*CYC1Sc*_, *HIS3* marker	This study
P_*GLK1B*_-EGFP	*K.m.* CEN/ARS, P_*GLK1B*_-EGFP-T_*CYC1Sc*_, *HIS3* marker	This study
P_*HSP*60_-EGFP	*K.m.* CEN/ARS, P_*HSP*60_-EGFP-T_*CYC1Sc*_, *HIS3* marker	This study
P_*TDH3*_-EGFP	*K.m.* CEN/ARS, P_*TDH3*_-EGFP-T_*CYC1Sc*_, *HIS3* marker	This study
P_*PGK*_-EGFP	*K.m.* CEN/ARS, P_*PGK*_-EGFP-T_*CYC1Sc*_, *HIS3* marker	This study
P_*HTB2*_-EGFP	*K.m.* CEN/ARS, P_*HTB2*_-EGFP-T_*CYC1Sc*_, *HIS3* marker	This study
P_*HTB1*_-EGFP	*K.m.* CEN/ARS, P_*HTB1*_-EGFP-T_*CYC1Sc*_, *HIS3* marker	This study
P_*HHF1*_-EGFP	*K.m.* CEN/ARS, P_*HHF1*_-EGFP-T_*CYC1Sc*_, *HIS3* marker	This study
P_*HHF2*_-EGFP	*K.m.* CEN/ARS, P_*HHF2*_-EGFP-T_*CYC1Sc*_, *HIS3* marker	This study
P_*TEF3*_-EGFP	*K.m.* CEN/ARS, P_*TEF3*_-EGFP-T_*CYC1Sc*_, *HIS3* marker	This study
P_*NC1*_-EGFP	*K.m.* CEN/ARS, P_*NC1*_-EGFP-T_*CYC1Sc*_, *HIS3* marker	This study
P_*PDC1*_-EGFP	*K.m.* CEN/ARS, P_*PDC1*_-EGFP-T_*CYC1Sc*_, *HIS3* marker	This study
P_*PDC1*_ (1400bp)- EGFP	*K.m.* CEN/ARS, P_*PDC1*_ (1400bp)- EGFP-T_*CYC1Sc*_, *HIS3* marker	This study
P_*PDC1*_ (1700bp)- EGFP	*K.m.* CEN/ARS, P_*PDC1*_ (1700bp)- EGFP-T_*CYC1Sc*_, *HIS3* marker	This study
P_*ADH1*_-2PS	*K.m.* CEN/ARS, P_*ADH1*_-2PS-T_*CYC1Sc*_, *HIS3* marker	This study
P_*PGK*_-2PS	*K.m.* CEN/ARS, P_*PGK*_-2PS-T_*CYC1Sc*_, *HIS3* marker	This study
P_*SSA3*_-2PS	*K.m.* CEN/ARS, P_*SSA3*_-2PS-T_*CYC1Sc*_, *HIS3* marker	This study
P_*HHF1*_–2PS	*K.m.* CEN/ARS, P_*HHF1*_–2PS-T_*CYC1Sc*_, *HIS3* marker	This study
P_*TEF3*_-2PS	*K.m.* CEN/ARS, P_*TEF3*_-2PS-T_*CYC1Sc*_, *HIS3* marker	This study
P_*NC1*_–2PS	*K.m.* CEN/ARS, P_*NC1*_–2PS-T_*CYC1Sc*_, *HIS3* marker	This study
P_Sc*ADH2*_-2PS	*K.m.* CEN/ARS, P_Sc*ADH2*_-2PS-T_*CYC1Sc*_, *HIS3* marker	This study
pKD-A2PS	*K. lactis* pKD1, P_Sc*ADH2*.c_-2PS-T_*CYC1Sc*_, *URA3* marker	McTaggart et al. (2019)
pKD-N2PS	*K. lactis* pKD1, P_*NC1*_–2PS-T_*CYC1Sc*_, *URA3* marker	This study

*Cloning and plasmid construction:* All plasmids used in this work are listed in Table 1, with primers summarized in Table S1 and promoter sequences summarized in Table S2. Primers (DNA oligos) were synthesized by Integrated DNA Technology (IDT). All promoter fragments were amplified from extracted yeast genome of *K. marxianus* CBS6556 *ΔHIS3 ΔURA3* by PCR using Q5® High-Fidelity DNA Polymerase purchased from New England BioLabs Inc (NEB). Expression plasmids for EGFP were created by Gibson assembly using NEBuilder® HiFi DNA Assembly Master Mix from NEB. Restriction enzymes and T4 ligase for construction of the majority of the 2-PS expression plasmids were purchased from NEB. All plasmids transformed in yeast were confirmed by Sanger sequencing from either Source BioScience or Genewiz.

For EGFP expression plasmid construction, the 700 bp long putative promoter of a given gene was identified and cloned from the genome of *K. marxianus* CBS6556 *ΔHIS3 ΔURA3* (see Table S1 for primers). Agarose gel (1%) electrophoresis was used to confirm and purify amplified promoter fragments from PCR. Fragments with the correct length recovered from gel were then inserted into a *K. marxianus* EGFP expression vector (pIW1198) by replacing *S*. *cerevisiae* P_*GPD1*_, the original promoter upstream of EGFP. The plasmid backbone was prepared by digestion with *Sac*II and *Xma*I for 1 ​h at 37 ​°C.

For construction of 2-pyrone synthase (2-PS) expression plasmids, pIW578 and pXP842-2PS (Table 1) were both digested with *Spe*I and *Xho*I and the 1233 bp fragment from pXP842-2PS was ligated to the linearized pIW578. Using *Spe*I and *Not*I restriction enzymes, *K. marxianus ADH1, HHF1, NC1, PGK1* and *SSA3* promoters and the *S. cerevisiae ADH2* promoter (P_Sc*ADH2*_) were then inserted upstream of 2-PS by replacing the *S. cerevisiae GPD1* promoter from pIW578-2PS with PCR amplicons from primer pairs FW-ADH1-2PS ​+ ​RV-ADH1-2PS, FW-HHF1-2PS ​+ ​RV-HHF1-2PS, FW-NC1-2PS ​+ ​RV-NC1-2PS, FW-PGK-2PS ​+ ​RV-PGK-2PS, FW-SSA3-2PS ​+ ​RV-SSA3-2PS, and FW-ADH2Sc-2PS ​+ ​RV-ADH2Sc-2PS respectively, using the corresponding EGFP-harboring vectors (P_*ADH1*_-EGFP, P_*HHF1*_-EGFP, P_*NC1*_-EGFP, P_*PGK*_-EGFP, and P_*SSA3*_-EGFP) and pKD-A2PS as templates. This resulted in the creation of P_*ADH1*_-2PS, P_*HHF1*_–2PS, P_*NC1*_-2PS, P_*PGK*_-2PS, P_*SSA3*_-2PS and P_Sc*ADH2*_-2PS. To generate P_*TEF3*_-2PS, the *Spe*I and *Not*I-digested pIW578-2PS plasmid was Gibson-assembled with the PCR product from primer pair FW-TEF3-2P​S ​+ ​RV-TEF3-2PS using P_*TEF3*_-EGFP as a template. This was necessary due to the presence of an internal *Spe*I restriction site in the selected *TEF3* promoter region. Finally to create pKD-N2PS, the *ADH2* promoter from *S. cerevisiae* was substituted by the *K. marxianus NC1* promoter in the high-copy plasmid pKD-A2PS (McTaggart et al., 2019) by introducing the PCR amplification product from primer pair FW-pKD1-NC1-2PS ​+ ​RV-pKD1-NC1-2PS using P_*NC1*_-2PS as a template and the restriction sites *Zra*I and *Spe*I.

*K. marxianus transformation:* CBS6556 *ΔHIS3 ΔURA3* cells were transformed using the protocol described by Löbs et al., 2017. Briefly: (1) Cells were cultured in 2 ​mL of 2% YPD overnight at 30 ​°C; (2) 400 ​μL of cell culture were harvested by centrifugation at 5000 ​rpm for 1 ​min; (3) harvested cells were washed with 1 ​mL of sterile water twice by resuspension and centrifugation at 5000 ​rpm for 1 ​min; (4)10 ​μL of carrier ssDNA (previously boiled for 5 ​min ​at 100 ​°C and chilled) were added to resuspend the cell pellets by gentle vortexing; (5) 500 ​ng of plasmid and 400 ​μL of transformation buffer (40% PEG, 0.1 ​M LiAc, 10 ​mM Tris-HCl, 1 ​mM EDTA, 70 ​mM DTT pH 7.5) were added and mixed well by pipetting; (6) the mixture was incubated at room temperature for 15 ​min, and heat-shocked for another 15 ​min ​at 47 ​°C. Cell pellets were then collected by centrifugation at 5000 ​rpm for 1 ​min and resuspended in 500 ​μL of selective media (SD-His). To obtain single colonies, 50 ​μL of the cell resuspension was plated in SD-His plates with 2% agar. KM1 Δ*URA3* cells were transformed via electroporation using an adaptation of the method described by (Benatuil et al., 2010) Briefly, (1) cells were grown overnight at 30 ​°C in two 250 ​mL baffled flasks containing 25 ​mL of YPD 2% each; (2) cell cultures were pooled and collected by centrifugation (2400 ​g, 3 ​min, 4 ​°C), (3) washed twice with 25 ​mL of iced-cold water and (4) once with 25 ​mL of ice-cold Electroporation Buffer (1 ​M Sorbitol, 1 ​mM CaCl_2_); (5) yeast cells were conditioned for 30 ​min ​at 30 ​°C in 10 ​mL of a 0.1 ​M LiAc/10 ​mM DTT solution and (6) then washed once again with 25 ​mL of ice-cold Electroporation Buffer; (7) the pellet was re-suspended in 1.6 ​mL of electroporation buffer and 400 ​μL of that solution were mixed with 1 ​μg of plasmid; (8) cells then electroporated in a 2 ​mm electrode gap cuvette (VWR) at 2.0 ​kV and 25 ​μF; (9) and then washed with 1 ​mL of pre-warmed SDCA media. A 1:2500 dilution of the cell suspension was plated on SDCA to obtain single colonies.

*Cell culture and growth curve measurements*. CBS6556 *ΔHIS3 ΔURA3* cells were inoculated from fresh SD-His plates into 1 ​mL of liquid SD-His and grown overnight at 30 ​°C. Cells were then transferred to 250 ​mL baffled flasks (initial OD_600_ ​= ​0.05) and grown until late stationary phase in 25 ​mL of SD-His. For each sample, the cell suspension was diluted 20 times and the optical density at 600 ​nm was measured using Nanodrop 2000c (Fisher Scientific) with a 1 ​cm light pathlength cuvette. For expression experiments in xylose, cells were inoculated from fresh SX-His plates (with 2% agar) into 1 ​mL of liquid SX-His and grown overnight at 30 ​°C. Cells were then transferred (initial OD_600_ ​= ​0.05) to fresh 1 ​mL of SX-His and cultivated for approximately 18 ​h. This culture was used as the inoculum for the experimental cultures in 25 ​mL of SX-His 250 ​mL baffled flasks (initial OD_600_ ​= ​0.05), which were grown until late exponential or late stationary phase. KM1 *ΔURA3* strains were cultivated as described by (McTaggart et al., 2019). Briefly, cells were inoculated from fresh plates into 3 ​mL of liquid 1% SXCA or 0.95% SLCA and grown overnight at 37 ​°C. Cells were then re-inoculated into 3 ​mL of fresh media at OD_600_ ​= ​0.1 and grown for 48 ​h ​at 37 ​°C in an orbital water bath shaker (Amerex Instruments, Inc., Model SK-929). For each sample, cells were diluted and optical density was measured at 600 ​nm in a Shimadzu UV-2450 spectrophotometer.

*EGFP thermal stability*. *K. marxianus* CBS6556 *ΔHIS3 ΔURA3* harboring an EGFP expression plasmid was cultured in SD-His medium at 30 ​°C with 0.05 initial OD_600_. Cells were harvested by 10 ​min centrifugation at 5000*g* after 14 ​h of culture. After washing twice with 40 ​mL PBS, cells were resuspended with 100 ​mL 1XPBS. After sonication, cell lysate was collected by centrifugation at 11,000 g for 30 min at 4 ​°C. The cell lysate was incubated at 30, 37 and 45 ​°C for different time. The fluorescence signal intensity of incubated lysate was measured at 0, 1, 2, 4, 6, 8, 16, 20 ​h using the BioTek®Synergy™ Neo2 multi-mode microplate Reader (excitation: 488 ​nm, emission: 511 ​nm).

*Dynamic EGFP fluorescence.* One hundred microliters of the cell suspension were transferred to a 96-well Nunc black plate (Thermo Scientific) and fluorescence was measured in a plate reader (SpectraMax M2, Molecular Devices, or BioTek Synergy™ Neo2 multi-mode microplate Reader) using an excitation wavelength of 488 ​nm and a 511 ​nm emission wavelength. For measurements using the BioTek Synergy™ Neo2 multi-mode microplate Reader, the cell suspension was diluted 20 times. For the EGFP fluorescence study, *K. marxianus* CBS6556 *ΔHIS3 ΔURA3* with blank plasmid (pIW578, see Table 1) was employed as the background of fluorescence intensity. All relative fluorescence intensity (RFU) shown in this study are background subtracted.

*Flow cytometry*. After 14 ​h of culture, cells were harvested by centrifugation at 5000 ​rpm, and washed twice with PBS. Samples were then diluted 50-fold in water and the fluorescence signal intensity per cell was determined (excitation wavelength: 488 ​nm, filter range: 533/30 ​nm).

*Determination of triacetic acid lactone levels.* Triacetic acid lactone concentrations were determined via HPLC-UV, as described by McTaggart et al. (2019), or spectrophotometrically using a plate reader. Control experiments were used to confirm that both methods provide the same results under our experimental conditions. For the HPLC-UV assay, samples were centrifuged at 2500×*g* for 5 ​min and 1 ​mL of the supernatant was collected and stored at 4 ​°C for further analysis. Twenty microliters of the samples were injected into an HPLC (Shimadzu, Japan) equipped with a UV–Vis detector (SPD-10 ​A VP; Shimadzu). A Zorbax SB-C18 reversed-phase column (2.1 ​Å~ 150 ​mm; Agilent Technologies, Santa Clara, CA) was used as a stationary phase. The mobile phase consisted of acetonitrile buffered in 1% acetic acid with a gradient of 95%–85% of water buffered in 1% acetic acid. This provided an elution time of approximately 12 ​min when the equipment ran at a flow rate of 0.25 ​mL/min and the temperature of the column was kept at 25 ​°C. For the spectrophotometric assay, the supernatant was diluted 20-fold and absorbance was measured in UV transparent flat bottom plate (Corning) using the plate reader at 277 ​nm (SpectraMax M3, Molecular Devices).

## 3 Results

**Fig. 1:**
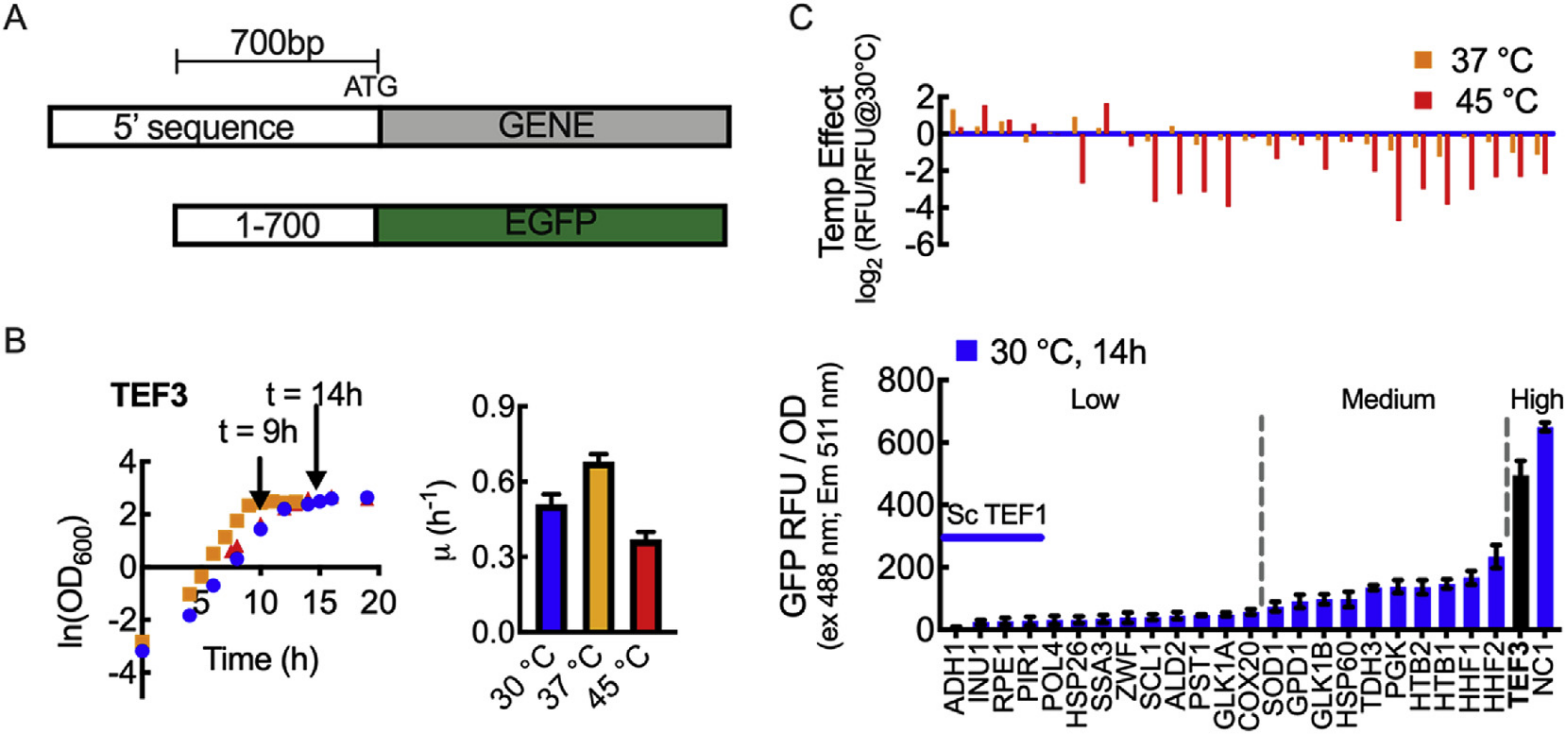
Screening and characterization of *K. marxianus* promoters for heterologous protein expression. (A) Twenty-five EGFP expression cassettes were constructed by identifying and cloning the 700 base pairs upstream of a start codon of a given *K. marxianus* gene. (B) Growth and growth rate of *K. marxianus* CBS6556 Δ*HIS3* Δ*URA3* in 2% glucose harboring a low copy number plasmid with EGFP expression driven by P_*TEF3*_. Shake flask cultures were inoculated with an initial OD_600_ of 0.05. Triplicate cultures were grown at 30, 37, and 45 ​°C. Arrows indicate the early stationary phase time points used for expression characterization across the promoter library. (C) Cell density normalized EGFP fluorescence (Relative Fluorescence Unit, RFU) at 30 ​°C for the promoter set. The P_*TEF3*_ result is shown in black for ease of reference to part (B). The effect of temperature (37 and 45 ​°C) is indicated above as the log_2_ fold change from fluorescence observed at 30 ​°C. In the lower panel, the strength of *S. cerevisiae TEF1* promoter is indicated by the horizontal blue line as a control. Vertical dashed lines show how promoters were grouped into low, medium and high strength sub-groups based on the EGFP RFU/OD at 30 ​°C. All data represent biological triplicates. Data points and bars indicate the mean; the error bars indicate the standard deviation. All RFU values are background substrate with CBS6556 *ΔHIS3 ΔURA3* harboring empty plasmid pIW578 as the background. (For interpretation of the references to colour in this figure legend, the reader is referred to the Web version of this article.)

All strains and plasmids used in this study are presented in Table 1. The plasmids include a set of vectors encoding EGFP with expression driven by one of 25 different promoters identified in *K. marxianus* CBS6556 *ΔHIS3 ΔURA3*. Promoters were selected by homology to the well-characterized *S. cerevisiae* genome and through previously published transcriptional analyses (Lee et al., 2015; Fu et al., 2019; Lertwattanasakul et al., 2015). Nine *K. marxianus* genes, *ADH1, HHF1, HHF2, HTB1, HTB2, PGK, THD3, TEF3* and *NC1* (putative homolog of *ScCCW12*), were identified by translated BLAST (tblastn) using protein queries from *S. cerevisiae*. These nine promoters already showed relatively high strength in *S. cerevisiae.* We assumed that similar promoter strength might also be observed in *K. marxianus.* Sixteen additional *K. marxianus* genes were selected because they were shown to be upregulated at 45 ​°C from reported transcriptomics data: *PST1, GLK1A, GLK1B, SCL1, ZWF, RPE1, GPD1, ALD2, HSP26, HSP60, PIR1, SSA3, SOD1, INU1, POL4*, and *COX20*. For each identified open reading frame (ORF), the 700 bp upstream of the start codon were cloned from the CBS6556 *ΔHIS3 ΔURA3* genome and inserted upstream of EGFP to construct the promoter screening library (Fig. 1A). The stability of EGFP assay indicated that EGFP was stable at 30, 37, and 45 ​°C for upward of 20 ​h (Fig. S1).

We elected to use only 700 bp for each promoter because a similar strategy was successful in creating native-derived promoters from *S. cerevisiae* sequences and because we wanted to minimize promoter length while maintaining core promoter and upstream activating sequence (UAS) function (Lee et al., 2015). We recognize that in some cases critical upstream regions may not have been incorporated within the tested sequences, thus resulting in expression level differences from the full-length native promoters. For example, truncation of the *K. marxianus PDC1* promoter (P_*PDC1*_) from 1400 bp down to 700 bp dramatically reduced expression (Fig. S2). Limiting sequences to 700 bp, however, was successful in creating a series of functional native-derived promoters. The *K. marxianus TEF3* promoter exemplifies the strategy; expression from the 700 bp *TEF3* promoter (P_*TEF3*_) was nearly 3-fold greater than full-length P_*PDC1*_ at 30 ​°C, which was previously identified as a high expression level promoter (Rajkumar et al., 2019).

With the promoter library in-hand, we first evaluated *K. marxianus* CBS6556 *ΔHIS3 ΔURA3* growth and EGFP expression during lag, exponential, and stationary phases with defined glucose media at 30, 37, and 45 ​°C. One example is illustrated in Fig. 1B, where growth curves and growth rates of CBS6556 *ΔHIS3 ΔURA3* with P_*TEF3*_-driven EGFP expression at various temperatures are shown. Growth rate was highest at 37 ​°C (0.7 h^−1^), with rapid growth also occurring at 30 and 45 ​°C (0.5 and 0.3 h^−1^, respectively). The growth curve and growth rate of CBS6556 *ΔHIS3 ΔURA3* harboring a blank plasmid was also determined in triplicate (Fig. S3). Similar growth rates in both conditions indicate that EGFP expression did not alter *K. marxianus* growth. Fig. 1C (bottom) shows the average fluorescence per OD_600_ for each promoter as cultures reached early stationary phase (t ​= ​14 ​h for 30 ​°C). Figs. S4 and S5 show equivalent plots for 37 and 45 ​°C cultures. Changes in promoter strength from data observed at 30 ​°C are shown in Fig. 1C (top). In the majority of cases, promoter expression decreased or stayed the same at higher temperatures in glucose media; however, P_*ADH1*_, P_*INU1*_, P_*PRE1*_, and P_*SSA3*_ resulted in higher expression as temperature increased. At 30 ​°C, P_*NC1*_ exhibited the highest expression level (as judged by relative EGFP fluorescence intensity), while P_*TEF3*_ was second highest. At the low end, 13 promoters (P_*ADH1*_, P_*INU1*_, P_*PRE1*_, P_*PIR1*_, P_*POL4*_, P_*HSP26*_, P_*SSA3*_, P_*ZWF*_, P_SCL1_, P_*ALD2*_, P_*PST1*_, P_*GLK1A*_ and P_*COX20*_) resulted in expression levels no more than 10% of P_*NC1*_. The remaining 10 promoters (P_*SOD1*_, P_*GPD1*_, P_*GLK1B*_, P_HSP60_, P_*TDH3*_, P_*PGK*_, P_*HTB2*_, P_*HTB1*_, P_*HHF1*_, and P_*HHF2*_) showed expression levels between the high and low sets. The relative groupings were found to be consistent at 37 ​°C, with the exception of P_*ADH1*_, which moved from the low to the medium-expression group (Fig. S4). At 45 ​°C, the relative groupings changed considerably. P_*SSA3*_, which had low expression at 30 and 37 ​°C, became one of the strongest promoters, eight of the thirteen weak promoters (*i.e.* P_*ADH1*_, P_*INU1*_, P_*PRE1*_, P_*PIR1*_, P_*POL4*_, P_*ZWF*_, P_*ALD2*_, and P_*COX20*_) joined the medium set, and the medium promoter P_*HTB2*_ moved to the low-expression set (Fig. S5). As shown in Fig. 1C, not all promoters that have previously been reported to be upregulated at 45 ​°C resulted in higher EGFP expression level. In our study, at least two key differences exist: (1) This study characterizes truncations of the native promoter, thus potentially altering function; and, (2) The production of EGFP, not the native gene associated with the promoter, removes potential post-transcriptional regulation that may be present during the transcriptional studies.

**Fig. 2:**
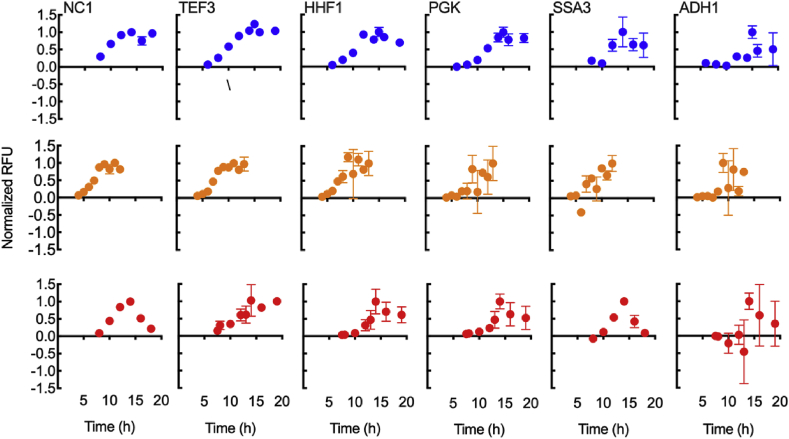
*K. marxianus* promoter dynamics using glucose as the carbon source. Time course data were collected from inoculation to stationary phase for 6 different *K. marxianus* promoters. Shake flask cultures were grown at 30 ​°C (blue), 37 ​°C (orange), and 45 ​°C (red) in synthetic defined media without histidine (SD-His) with 2% glucose. All data is normalized to the highest fluorescence signal intensity for each combination of promoter and temperature. The promoter strength upon reaching stationary phase is shown in Fig. 1. Data points represent the mean of biological triplicates, while the error bars represent the standard deviation. (For interpretation of the references to colour in this figure legend, the reader is referred to the Web version of this article.)

From the complete list of 25 native-derived promoters, we selected the following six as a defined promoter set representing a broad range of expression levels: P_*NC1*_ and P_*TEF3*_ were classified as strong promoters, P_*HHF1*_ and P_*PGK*_ were grouped as medium, and P_*ADH1*_ and P_*SSA3*_ were defined as weak. These groupings were based on expression levels measured at 30 ​°C, but the same set of promoters also provides a broad range at both 37 and 45 ​°C. The dynamics of each of the six promoters of the defined set is shown in Fig. 2 for glucose cultures at 30, 37, and 45 ​°C. Overall, it is apparent that expression increased through exponential phase, reaching the maximum at early stationary phase. In most cases, EGFP fluorescence plateaued through the stationary phase until the end of the measurement period. It is important to note that expression levels are reported with background fluorescence subtracted and expression levels resulting from P_*ADH1*_ and P_*SSA3*_ at 30 ​°C are low but significantly above the background. Comparison within each group over different temperatures shows that expression from P_*NC1*_, P_*TEF3*_, P_*HHF1*_, and P_*PGK*_ decreased with increasing temperature, while P_*SSA3*_ and P_*ADH1*_ had improved performance at higher temperature. We also note here that the expression of P_*SSA3*_ and P_*ADH1*_ appeared to decrease at longer times at elevated temperature. These two promoters represent genetic parts that could be further optimized for high-level expression at the upper limit of *K. marxianus* thermotolerance.

**Fig. 3:**
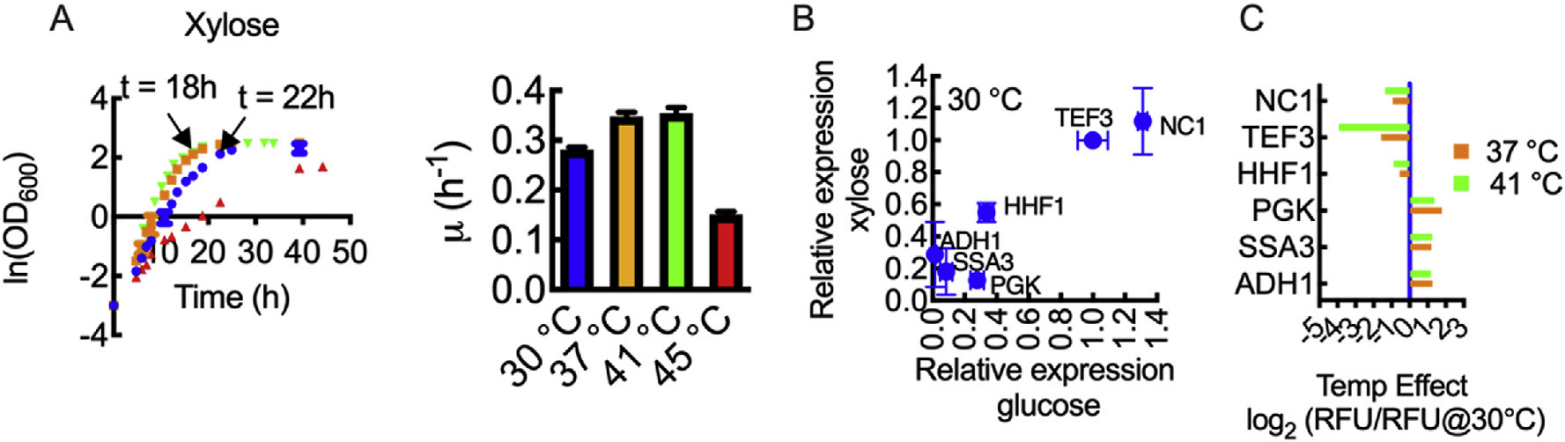
Characterization of six selected promoters at late exponential phase when grown on xylose. (A) Growth curves and growth rate of *K. marxianus* CBS6556 *ΔHIS3 ΔURA3* harboring a blank expression vector (pIW578) in synthetic defined media with 2% xylose as a carbon source at 30, 37, 41 and 45 ​°C. Shake flask cultures with an initial OD_600_ of 0.05 were inoculated with starter cultures grown on xylose. (B) Comparison of relative promoter strength with growth on xylose and glucose at 30 ​°C. EGFP fluorescence was normalized to P_*TEF3*_ for xylose and glucose separately. The absolute value of RFU/OD for each promoter is 25 ​± ​18 (*ADH1*), 47 ​± ​7 (*HHF1*), 95 ​± ​14 (*NC1*), 10 ​± ​1 (*PGK*), 15 ​± ​11 (*SSA3*) and 86 ​± ​3 (*TEF3*) at 30 ​°C. (C) The temperature effect on promoter strength with xylose as a carbon source. The effect of temperature (37 and 41 ​°C) is indicated as the log_2_-fold change from fluorescence observed at 30 ​°C. Time-points for late exponential phase in xylose are 22 ​h ​at 30 ​°C and 18 ​h ​at 37 ​°C and 41 ​°C. All data represent biological triplicates. Data points and bars indicate the mean and error bars indicate the standard deviation.

The data thus far shows that there is a strong effect of temperature on promoter activity. It is also well-known that promoter strength can vary with carbon source (Weinhandl et al., 2014; McTaggart et al., 2019; Rajkumar et al., 2019); thus, we evaluated the six promoters in our expression set using xylose as a carbon source. Fig. 3A presents growth curves for CBS6556 *ΔHIS3 ΔURA3* harboring control plasmid pIW578 in synthetic defined media with 2% xylose at 30, 37, 41, and 45 ​°C. The additional 41 ​°C data was collected due to a dramatically slower growth rate at 45 ​°C with respect to that observed when cells were grown in 2% glucose (0.3 h^−1^ with glucose at 45 ​°C, 0.14 h^−1^ with xylose at 45 ​°C). The growth rates on xylose at 30, 37, and 41 ​°C were significantly higher than at 45 ​°C (0.28 ​h^−1^ ​at 30 ​°C, 0.34 ​h^−1^ ​at 37 ​°C, and 0.35 ​h^−1^ ​at 41 ​°C), but overall the results indicate that *K. marxianus* CBS6556 *ΔHIS3 ΔURA3* has slower growth on xylose than on glucose. In all subsequent xylose experiments, the highest temperature investigated was 41 ​°C.

With respect to promoter function, we directly compared EGFP expression with P_*TEF3*_ at 30 ​°C in 2% glucose and 2% xylose. Flow cytometry data suggests that for P_*TEF3*_, carbon source has little to no effect on protein production (Fig. S6); as such we normalized expression in glucose and xylose to the P_*TEF3*_ expression level (Fig. 3B). Comparison of the rank order of the six promoters in both xylose and glucose suggests that this is also true for P_*NC1*_, P_*HHF1*_ and P_*SSA3*_. The *NC1* promoter resulted in the highest expression level, while P_*HHF1*_ and P_*SSA3*_ can be considered medium- and low-level promoters, respectively for both carbon sources at 30 ​°C. Variation in the rank order of promoters (and in absolute expression level) was observed in the lower range. In glucose, P_*PGK*_ was found to be a medium level promoter, reaching 28% of P_*TEF3*_, but in xylose expression was reduced to less than 12% of P_*TEF3*_. Growth in xylose had the opposite effect on P_*ADH1*_, increasing expression to 28% of P_*TEF3*_.

The temperature effect was also studied with 2% xylose and the corresponding data is shown in Fig. 3C. The results indicate that the three promoters with highest expression levels at 30 ​°C (*i.e.*, P_*NC1*_, P_*TEF3*_ and P_*HFF1*_) show reduced EGFP fluorescence at higher temperatures. In contrast, the lower range promoters (*i.e.*, P_*PGK*_, P_*ADH1*_ and P_*SSA3*_) showed between 2.7 and 3.5-fold increase in expression at 37 and 41 ​°C.

**Fig. 4:**
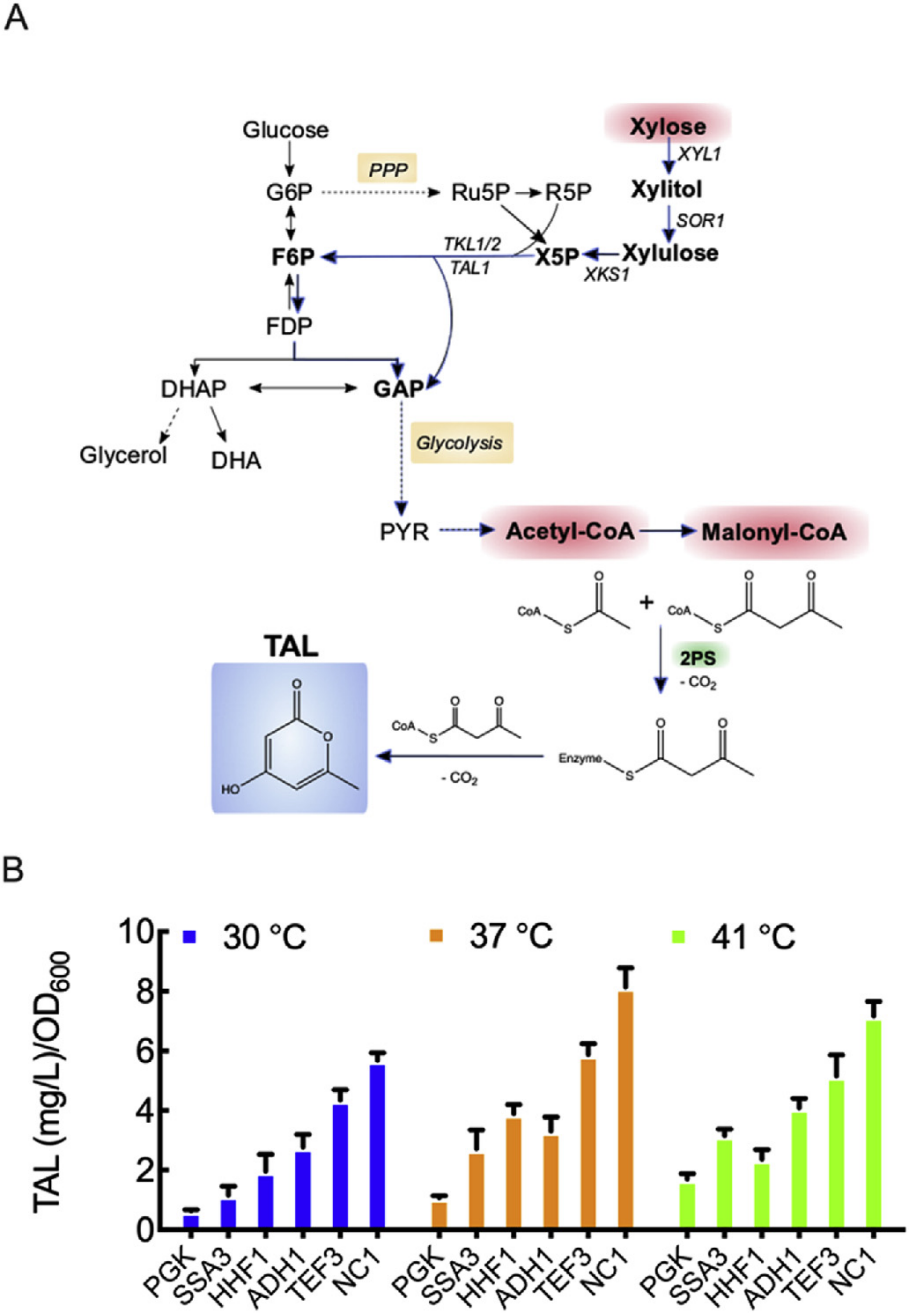
Triacetic acid lactone (TAL; 4-hydroxy-6-methyl-2-pyrone) biosynthesis in xylose medium with varying levels of 2-pyrone synthase (2-PS) expression. (A) Simplified illustration of proposed TAL biosynthesis pathway in *K. marxianus.* (B) Specific TAL production at late exponential phase at 30, 37 and 41 ​°C. 2-PS was overexpressed with *PGK, SSA3, HHF1, ADH1, TEF3*, and *NC1* promoters on a low copy number plasmid. All data represent biological triplicates and the error bars indicate the standard deviation.

To demonstrate the utility of the defined promoter set, we engineered triacetic acid lactone (TAL) biosynthesis in *K. marxianus* (Fig. 4A). Our previous work demonstrated that *K. marxianus* is capable of achieving high TAL titers using low cost carbon substrates such as glycerol and xylose, without metabolic pathway engineering (McTaggart et al., 2019). TAL is a valuable platform chemical that can be derivatized into both high-value and commodity chemicals, including direct substitutes of current petrochemicals such as dienoic acid and sorbic acid, among others (Chia et al., 2012). Biological synthesis of TAL is catalyzed by the type III polyketide synthase 2-pyrone synthase (2-PS), natively found in the plant *Gerbera hybrida* (Eckermann et al., 1998). The choice of promoter controlling the synthesis of 2-PS has been shown to be an important parameter for optimal TAL production (Cardenas and Da Silva, 2014; McTaggart et al., 2019). We therefore decided to test the effect of promoter strength (P_*ADH1*_, P_*HHF1*_, P_*NC1*_, P_*PGK*_, P_*SSA3*_ and P_*TEF3*_) on TAL specific titers at different temperatures using xylose as the carbon source. The six promoters were placed upstream of the *G. hybrida* 2-PS gene in a low copy number plasmid and transformed into CBS6556 *ΔHIS3 ΔURA3*. We kept our expression system consistent to allow comparisons with the fluorescence-based measurements and for consistent plasmid copy number. The resulting strains were grown in synthetic defined medium with 2% xylose at 30, 37, and 41 ​°C, and TAL levels were measured at late exponential phase and late stationary phase (Fig. 4B and S7, respectively).

The six promoters tested resulted in a wide range of TAL specific titers, covering a 17.8-fold change between the highest and the lowest measured across all temperatures and promoters. Most promoters showed higher levels of TAL as temperature increased from 30 to 37 ​°C, except for P_*ADH1*_, which did not show statistically significant changes with temperature. When temperature was increased from 37 to 41 ​°C, none of the six promoters resulted in a further increase in TAL production (Fig. 4B and S7). These general trends are in accordance to those reported in our earlier work (McTaggart et al., 2019) when expressing 2-PS under the control of the full-length P_*PGK*_; higher TAL specific titers were achieved as temperature increased from 30 to 37 ​°C, but remained constant when temperature was further increased.

For the three temperatures tested, 2-PS expression controlled by P_*PGK*_ and P_*NC1*_ resulted in the lowest and the highest TAL specific titers measured, respectively. Therefore, if TAL titers are taken as a proxy for promoter strength, P_*PGK*_ would be the weakest and P_*NC1*_ the strongest promoter. At 30 ​°C, P_*SSA3*_ showed comparable levels to P_*PGK*_, but at higher temperatures it behaved as a medium-strength promoter. P_*ADH1*_ and P_*HHF1*_ performed as medium-strength promoters across temperatures and P_*TEF3*_ acted as a strong promoter at 37 and 41 ​°C.

In our previous work, the *ADH2* promoter from *S. cerevisiae* (P_Sc*ADH2*_) outperformed the native *K. marxianus PGK1* promoter when placed upstream of 2-PS (McTaggart et al., 2019). We thus decided to compare TAL production levels between our strongest promoter P_*NC1*_ and P_Sc*ADH2*_ using our current expression system (*i.e.*, on a CEN/ARS plasmid in strain CBS 6556 *ΔHIS3 ΔURA3*) at 37 ​°C. No statistically significant difference in titers or specific titers were observed between the two promoters (Fig. S8) at late exponential or stationary phase; therefore, the *K. marxianus NCI* promoter was comparable to the strong *S. cerevisiae ADH2* promoter for TAL synthesis. TAL titers ranged from 58 ​mg/L to 64 ​mg/L at late exponential phase and from 94 to 114 ​mg/L in stationary phase. These low titers are expected when using a low-copy plasmid in strain CBS 6556 *ΔHIS3 ΔURA3*.

McTaggart et al. reported their highest TAL titer at 48 ​h when 2-PS was expressed from a multi-copy plasmid under the control of the P_Sc*ADH2*_ in the industrial strain KM1 Δ*URA3*. To further compare the performance of P_*NC1*_ and P_Sc*ADH2*_, we replaced P_Sc*ADH2*_ in pKD-A2PS with P_*NC1*_ to create pKD-N2PS. We then transformed both plasmids into KM1 Δ*URA3* and measured TAL production at 37 ​°C in 1% SXCA at 48 ​h (Fig. S9). Titers for KM1 Δ*URA3* pKD-A2PS and KM1 Δ*URA3* pKD-N2PS were 1.2 ​g/L and 0.82 ​g/L, respectively, the former being consistent with our previously reported value (McTaggart et al., 2019). While the use of P_*NC1*_ resulted in a 34% decrease in titer, there was no statistically significant difference between specific titers. Interestingly, when the same strains were tested using lactose as a carbon source, the use of P_*NC1*_ resulted in a 58% increase in titer and an 80% increase in specific titer (Fig. S9). Taken together, these results indicate that the use of P_*NC1*_ provides equivalent or higher TAL specific titers than P_Sc*ADH2*_.

## 4 Discussion

In this work, we set out to design and characterize a set of promoters for controlling gene expression in the thermotolerant yeast *K. maxianus*. From a set of 25 genes, we created a plasmid library for heterologous expression with native-derived promoters derived from *K. marxianus* sequences. These native-derived promoters were created from the 700 bp upstream of the start codon of each gene (Fig. 1A). Heterologous EGFP expression with glucose as the carbon source helped define a subset of six promoters with a broad transcriptional range (Fig. 2). We then chose a defined promoter set that includes P_*NC1*_, P_*TEF3*_, P_*HHF1*_, P_*PGK*_, P_*SSA3*_ and P_*ADH1*_ and is representative of the full 25-member library including promoters with high, medium, and low expression levels. These six promoters also maintained substantial transcriptional differences under xylose metabolism (Fig. 3B). We note that the *ADH1* promoter reported by Yang et al. (2015) showed a different temperature dependence to the *ADH1* promoter in our study. The difference was likely due to: (1) differences in *ADH1* promoter sequence (one sequence was from CBS 6556, while the other was from NBRC 1777); (2) differences in the studied strain (CBS 6556 vs. NBRC1777); and, (3) in our study we use EGFP as a proxy for transcriptional strength the function of which is invariant with temperature (Fig. S1), whereas Yang et al. used β-glucuronidase activity as a reporter, but the relationship between enzyme function and temperature was not described.

Our studies on TAL production from xylose confirmed the broad transcriptional range and utility of the library. Specifically, relative promoter strengths for 2-PS expression, a necessary heterologous reaction step in TAL biosynthesis, matched the relative EGFP expression from the same promoter set at 30 ​°C (Fig. S10A). The consistency between the rank order of promoter strength judged by TAL production and separately by EGFP fluorescence was lost at 37 and 41 ​°C (Fig. S10). One likely reason is that TAL biosynthesis does not rely solely on the expression of 2-PS, but also on the expression of upstream enzymes that may or may not change at high temperature. This hypothesis is supported by transcriptional studies in glucose, which have shown the *ACC1* gene is up-regulated at higher temperatures while *CIT1* is down-regulated (Fu et al., 2019; Lertwattanasakul et al., 2015). Thus acetyl-CoA and malonyl-CoA pools also are likely affected by temperature and TAL titers may not necessarily correlate exclusively with levels of 2-PS protein.

A valuable trait of *K. marxianus* is its thermotolerance, the benefit of which is observable for TAL biosynthesis; specific TAL titers at 37 and 41 ​°C were significantly greater than those achieved at 30 ​°C (Fig. 4). However, the production benefits realized at elevated temperature must be balanced with increased cellular stress. High growth rates on xylose were possible at 30, 37 and 41 ​°C, but growth rate was reduced by 2 to 3-fold at 45 ​°C. Different behavior was observed when glucose was used as the carbon source as high growth rate was maintained up to 45 ​°C. While there have been a number of recent studies focusing on the effects of temperature on *K. marxianus* transcription (Fu et al., 2019; Lertwattanasakul et al., 2015), the global effects on metabolism and promoter function have yet to be fully understood.

## 5 Conclusions

The results presented here help advance *K. marxianus* as a thermotolerant host for chemical biosynthesis. The primary result is the design and validation of a new set of promoters that can be used to vary gene expression by upward of 87-fold under glucose metabolism and greater than 17.8-fold with xylose as the carbon source. The defined set includes P_*NC1*_ and P_*TEF3*_ as strong promoters, P_*HHF1*_ and P_*PGK*_ as medium level promoters, and P_*SSA3*_ and P_*ADH1*_ as low-expression promoters. The temperature effect on gene expression from a subset of six promoters was also characterized, and showed that, in general, fluorescence was reduced as temperature increased from 30 ​°C to 37, 41, and 45 ​°C. Two promoters, P_*SSA3*_ and P_*ADH1*_, were exceptions to the trend and were found to have a positive correlation with temperature in both glucose and xylose. We demonstrated the utility of the promoter set by expressing 2-PS for TAL biosynthesis from xylose and showed increased TAL specific titers at 37 and 41 ​°C. Finally, when the strongest promoter P_*NC1*_ was tested in the industrial strain KM1 Δ*URA3* using a multi-copy plasmid in xylose and lactose, specific TAL titers were equivalent or higher than those using the previously reported heterologous P_Sc*ADH2*_.

## CRediT authorship contribution statement

**Xuye Lang:** Conceptualization, Methodology, Validation, Investigation, Data curation, Writing - original draft, Writing - review & editing, Visualization. **Pamela B. Besada-Lombana:** Methodology, Validation, Investigation, Data curation, Writing - original draft, Writing - review & editing, Visualization. **Mengwan Li:** Methodology, Validation, Investigation, Data curation, Writing - original draft, Writing - review & editing, Visualization. **Nancy A. Da Silva:** Conceptualization, Methodology, Validation, Investigation, Resources, Data curation, Writing - original draft, Writing - review & editing, Visualization, Supervision, Project administration, Funding acquisition. **Ian Wheeldon:** Conceptualization, Methodology, Validation, Investigation, Resources, Data curation, Writing - original draft, Writing - review & editing, Visualization, Supervision, Project administration, Funding acquisition.
