# Supplementary material for: Developing a broad-range promoter set for metabolic engineering in the thermotolerant yeast Kluyveromyces marxianus
Source: Metab Eng Commun. 2020 Sep 3;11:e00145. doi: 10.1016/j.mec.2020.e00145 (PMC7508702; doi:10.1016/j.mec.2020.e00145)
Supplement: Multimedia component 1 [file mmc1.docx]

**Supplementary Material**

**Developing a broad-range promoter set for metabolic engineering in the thermotolerant yeast *Kluyveromyces marxianus***

 Xuye Lang^1†^, Pamela B. Besada-Lombana^2†^, Mengwan Li^1^, Nancy A. Da Silva^2‡^and Ian Wheeldon^1,3‡^

1.  Department of Chemical and Environmental Engineering, UC Riverside

2.  Department of Chemical and Biomolecular Engineering, UC Irvine

3.  Center for Industrial Biotechnology, UC Riverside

^†^ Equal Contribution

^‡^Corresponding Author

Table S1: Summary of primers used in this study

| Primers | Sequence |
| --- | --- |
| FW-ADH1-GFP | GGGGTCCAATACCACCCGCCCATCATCCATCCACACACGC |
| RV-ADH1-GFP | CACCAGTCATGCTAGCCATCCCGGGTGTGTTGTGTATATGATTTTGTT |
| FW-INU1-GFP | GGGGTCCAATACCACCCGCCATGGAAAAAAATGATGTTGA |
| RV-INU1-GFP | CACCAGTCATGCTAGCCATCCCGGGATCTAACAAAAAAAAAATTAAATGT |
| FW-PRE1-GFP | GGGGTCCAATACCACCCGCCTGGGCAACTTCTCAAACCCATG |
| RV-PRE1-GFP | CACCAGTCATGCTAGCCATCCCGGGGCACAGCTAGAAACTGGAAAACCAC |
| FW-PIR1-GFP | GGGGTCCAATACCACCCGCCTGGGTTTTCAAATTGACATT |
| RV-PIR1-GFP | CACCAGTCATGCTAGCCATCCCGGGTGTATAAATTGAAATGTTTGGATTG |
| FW-POL4-GFP | GGGGTCCAATACCACCCGCCAATAACAAGTAAGTGCAATGCTAAA |
| RV-POL4-GFP | CACCAGTCATGCTAGCCATCCCGGGTGTACCTCATCGAACCCTTG |
| FW-HSP26-GFP | GGGGTCCAATACCACCCGCCCATGACAAGGGTGCATGTCC |
| RV-HSP26-GFP | CACCAGTCATGCTAGCCATCCCGGGCTCGTAATCGCTTTTGTTCTTAGTT |
| FW-SSA3-GFP | GGGGTCCAATACCACCCGCCTTGCCACTATCAAGTGAGCG |
| RV-SSA3-GFP | CACCAGTCATGCTAGCCATCCCGGGAATTCTTCTTCTTTTTTAGCTCTGT |
| FW-ZWF-GFP | GGGGTCCAATACCACCCGCCTAAAAAAAAAGGGTGGGATTGC |
| RV-ZWF-GFP | CACCAGTCATGCTAGCCATCCCGGGCTTGTTCAAATGTTCAGTATTATGT |
| FW-SCL1-GFP | GGGGTCCAATACCACCCGCCAGGCTTGCGCTTCTCAAACA |
| RV-SCL1-GFP | CACCAGTCATGCTAGCCATCCCGGGTCTGTTCACACTTGATAACGCTC |
| FW-ALD2-GFP | GGGGTCCAATACCACCCGCCCTCTAAAACCCATGTCGCAT |
| RV-ALD2-GFP | CACCAGTCATGCTAGCCATCCCGGGATTTCTACTTTTGTATACGGCTGAT |
| FW-PST-GFP | GGGGTCCAATACCACCCGCCCATTTCAGAGTTCTTTTGTC |
| RV-PST-GFP | CACCAGTCATGCTAGCCATCCCGGGATCTGTTAATATGAACAGGT |
| FW-GLK1A-GFP | GGGGTCCAATACCACCCGCCCCTTCACAAAAGAAAAGTATTTTGT |
| RV-GLK1A-GFP | CACCAGTCATGCTAGCCATCCCGGGATTTAAGTTTTATGTTACTAGTTTC |
| FW-COX20-GFP | GGGGTCCAATACCACCCGCCGAAAATTGTCATAAACGTTCCTCTG |
| RV-COX20-GFP | CACCAGTCATGCTAGCCATCCCGGGTATGTACACCAATCTCCAGG |
| FW-SOD1-GFP | GGGGTCCAATACCACCCGCCAGAATATTTTTTTTTTTTTTTGTTT |
| RV-SOD1-GFP | CACCAGTCATGCTAGCCATCCCGGGGATTAATTAATTGTATGTGTTATGT |
| FW-GPD1-GFP | GGGGTCCAATACCACCCGCCAAAGGGACAATCGGAGAATA |
| RV-GPD1-GFP | CACCAGTCATGCTAGCCATCCCGGGATCTGAAAACTGATTCCTCC |
| FW-GLK1B-GFP | GGGGTCCAATACCACCCGCCTTGCTAGCCGTTTTTCCAAG |
| RV-GLK1B-GFP | CACCAGTCATGCTAGCCATCCCGGGTTTTGTAAGTGTGTGTGTTTGTAAT |
| FW-HSP60-GFP | GGGGTCCAATACCACCCGCCTTAGCTAGTGTACAAACAT |
| RV-HSP60-GFP | CACCAGTCATGCTAGCCATCCCGGGTGTATTGTATTGTATTATATTAATT |
| FW-TDH3-GFP | GGGGTCCAATACCACCCGCCCACTATATCAGGCCTCCACT |
| RV-TDH3-GFP | CACCAGTCATGCTAGCCATCCCGGGTGTGAATGTGTAAAAGTGTGTGT |
| FW-PGK-GFP | GGGGTCCAATACCACCCGCCTTACCCTCACTCTTTCACAT |
| RV-PGK-GFP | CACCAGTCATGCTAGCCATCCCGGGTTTTGTATCTTTATATAGGTAGTG |
| FW-HTB2-GFP | GGGGTCCAATACCACCCGCCATGCCCAAACCCCGTGCCAT |
| RV-HTB2-GFP | CACCAGTCATGCTAGCCATCCCGGGTGTGTATGTATATGTGATTTAGTTT |
| FW-HTB1-GFP | GGGGTCCAATACCACCCGCCTATTATGTTGTGTTTATGTTATG |
| RV-HTB1-GFP | CACCAGTCATGCTAGCCATCCCGGGTTTTTCTGTATTATTTGGTTATC |
| FW-HHF1-GFP | GGGGTCCAATACCACCCGCCGTAAGTAATGGAGGATTGATATG |
| RV-HHF1-GFP | CACCAGTCATGCTAGCCATCCCGGGGTTTATTTATTGATTGCTGTTTA |
| FW-HHF2-GFP | GGGGTCCAATACCACCCGCCCTGGCAGGTGGTTGGACGGTCATTTA |
| RV-HHF2-GFP | CACCAGTCATGCTAGCCATCCCGGGGTTTGTTTATTGATTGTTTGTTGC |
| FW-TEF3-GFP | GGGGTCCAATACCACCCGCCAACACCGATGAAGCAAAGAA |
| RV-TEF3-GFP | CACCAGTCATGCTAGCCATCCCGGGCTTTAATGTTACTTCTCTTGGAG |
| FW-NC1-GFP | GGGGTCCAATACCACCCGCCCCACGCAGTGTGAATGGACTTT |
| RV-NC1-GFP | CACCAGTCATGCTAGCCATCCCGGGTTTTGATTTGTGTTTAAGCGAGTGA |
| FW-PDC1-GFP | TATGGGGTCCAATACCACCCATAAGAGTGGGCGTTGCACA |
| RV-PDC1-GFP | CACCAGTCATGCTAGCCATCCCGGGTGCAATTATTTGGTTTGGGTGTGG |
| FW-1400bpPDC1-GFP | TATGGGGTCCAATACCACCCCCTCCACAACTTTGTCCCCG |
| RV-1400bpPDC1-GFP | CACCAGTCATGCTAGCCATCCCGGGTGCAATTATTTGGTTTGGGTGTGG |
| FW-1700bpPDC1-GFP | TATGGGGTCCAATACCACCCAACGACGAGGTAAACAAGCTAAGAC |
| RV-1700bpPDC1-GFP | CACCAGTCATGCTAGCCATCCCGGGTGCAATTATTTGGTTTGGGTGTGG |
| FW-ADH1-2PS | GTGGCGGCCGCCATCATCCATCCACACACGCACA |
| RV-ADH1-2PS | CCCATACTAGTTTGTGTTGTGTATATGATTTTGTTTTTAAATGTAGTAACC |
| FW-HHF1-2PS | TGGCGGCCGCGTAAGTAATGGAGGATTGATATGTTCTTGTTC |
| RV-HHF1-2PS | TCCCATACTAGTGTTTATTTATTGATTGCTGTTTATTGCTTGTTG |
| FW-NC1-2PS | TGGCGGCCGCCCACGCAGTGTGAATGGACTTTC |
| RV-NC1-2PS | TCCCATACTAGTTTTTGATTTGTGTTTAAGCGAGTGACTGAAG |
| FW-PGK-2PS | TGGCGGCCGCTTACCCTCACTCTTTCACATTACC |
| RV-PGK-2PS | ATCCCATACTAGTTTTTGTATCTTTATATAGGTAGTGTGTATTAAAATGTATC |
| FW-SSA3-2PS | TGGCGGCCGCCTTGCCACTATCAAGTGAGCG |
| RV-SSA3-2PS | ATCCCATACTAGTAATTCTTCTTCTTTTTTAGCTCTGTAAAAATTC |
| FW-TEF3-2PS | GCGAATTGGAGCTCCACCGCGGTGGCGGCCGCAACACCGATGAAGCAAAGAAGTAACAG |
| RV-TEF3-2PS | GATGATGATGATGCATACTAGTCTTTAATGTTACTTCTCTTGGAGTTAGAACTATG |
| FW-ADH2Sc-2PS | TTTAGCGGCCGCAAAACGTAG |
| RV-ADH2Sc-2PS | GGCCACTAGTTATTACGATATAGTTAATAGTTGATAGTTGATTG |
| FW-PKD1-NC1-2PS | AAAAGACGTCCCACGCAGTGTGAATGG |
| RV-PKD1-NC1-2PS | TTTTACTAGTTTTTGATTTGTGTTTAAGCGAGTGACTG |

Table S2. Summary of promoter sequence

| Promoter | Promoter sequence (5’-3’) |
| --- | --- |
| ADH1 | CATCATCCATCCACACACGCACACGGCTTCCTGTCCTCCTCTATCCCCGGCACAGAAAATCCTGGAGAATGCCACTGCTTCTGCCCCCCCGCACTTACTGCGGAAAATCTGCCCATATGCCCATATGCCCATATATGCCTGGGCTTGCCTCAGGCAAGCCCAGGCGTACGTACAGACGAATCCAGCACGAATCCAGCACGAATCCACTATACACCACCCAGAGTAAAGGATCGTGATTCGAATTTTTTATTTTGCTAGGCACTTCGCGGCACTTCGCTTGGTGAGTGTGATTCGATTCCATCATTCCTGTTGTTTTTTCTATTTTGTCGCATTGCTGCTTGCTTGCTTGCGGGATGCCTGTTGCATCATCGTATGCCCCTAATTATAGCTTCCACAACTCGAAAGTGGAATCCAGTCCGGTCCTGCCCCAGATAGACGCTGCCTGCTGCCTGCCGCCTGCCGCCCTGCTTCCCTTTTTTCAGTTCAGTTGGAAATTGGAAATGTGAATTTTAGGAAATTTCTCTGTATAAAAGGGGGCACTTCGAGCGCTGAAGTATCTTCATCTGGAGTATACCTTTTATTAATTATTATTACTGTTGTTGCTATTACTATTGTAATACTTTTTATTTCCCTCCCCAAGCACAACTTAAATTAAGTAAGTGGTTACTACATTTAAAAACAAAATCATATACACAACACA |
| INU1 | ATGGAAAAAAATGATGTTGATGTTGAGTTAGTTGGGTTGAGTCAATTAGTGCGTGAAAGTATCACCACTTTTGTCATCCGGCGTTTCTGTGCGAATCACACACACACACACACACAGTTTATTGGAGCGCTTGTTTCTGGCGTATTCGTAATTGTTCTGCGGTGCGGTTCTGTGTGCATTTTTCCTGGGGTGTCTGCCGCACCTACTCATCACCCACGCCGTGGGTTTGAGCCATGGCGGAGGTACGACTGACTGGCTGCCTGCCTGCCTGACTGACTGCCTGACTGCAGGAAAAGAGGGTTTCGAAGGAAAAACTTTTCCTGTGTTAATCCGGCCGTGCGCCGCTGCTCCAAAATCCATCTTCATGAGAAGGAGTTTGAAAAAACAAAAAAATTCACATATAAAAAGCGTATCTCGAGATCTCAAAGTCTCCCTTGAATCGTGTTTGCCAGTTGTAACTCATCCTTTATTCTTCTATTCTATCTCTCTCTTTCCTTCCCCTAATCAGCAATTAAATCCGGGGTAAGGAAGAATTACTACTGTGTGTAACGGTTATATTTCGTTTTTTATTTTTTTTTCCATTGCCATAGAGAAAGAAAAAAAAAAAAAGAGAGTTTGTGAAGATCTTCCATTCGAATCCCATAAGTGACACATTTAATTTTTTTTTTGTTAGAT |
| PRE1 | TGGGCAACTTCTCAAACCCATGCTCCTCATCAAATTCCGCATCCTCGCTTTCCTCGTCAATGTTCAACACTTCAAGCTTCTCGTTTATTCTCGACAACGATGGACTCTCATTGTACTGCGATTCATCTTCCTGATCCTGCTCCTGTTGCTTCTTCTTCAAATACGAATCCTTCAACACACTCGCTGGTGTGTTGTGAGGAGTCGGAGCATTATCTATTTTGCTTGATCTCGTCGTCTTAATAGGCCTAGACATTGCTAATTTAAAACACTCTACAGCCTCAATACCCTATCTACCTTTTAAACCGGGTACCAAAATTACCCTATAATCCAAACACGTCTCTACTAAGCACCCCCAAAGTGGTGTGCTGTTGAACTACTCTTTACTAATGCTCAAAAAATTTTTATGTACGGGTTCCTATTTACTACCATATGTATGTTCTGAAAATACAGTATAATAGGCTAAACTATTCCTAAAATAAGACAAAACATATACAAACAGACATCTCCACTCGGGAGTTCCAGGAACTAGCAGCATGTACAGACAGACACGCACACAGAGTGTGCACAGCTGTTATCCAAGTAGGCAATACAACAAATACTCTGTGTACTAAGTACTACTCTTGTCATGTTTTCCGGTTCTATGTATCTATGCATAGTATAATATTATTCGGCCTCGTGGTTTTCCAGTTTCTAGCTGTGC |
| PIR1 | CTGGGTTTTCAAATTGACATTAATTTTTTTAACCAGCTTGAAATTTCATGGTGCGAATATTCTGTACGGAAAAAAAAAAAAATACACACAAAGCACATACAGATCTACGTAGGACGAAAGGTACCAAAGGGGAACGAGTGGCTGGTTGTGGTTTATTTTCCCTCAGTCACTCTTCTTGAGTTGAGTTCTGTCGAGCTCTATTGTCCGATGATTACCTTTTCCTCAGTATTCATTGATCAAAGAGAGGCTAATGCTACTATTTCTAATTTTTTTGCTGGGATCAATATATCGAATTCGGCAATTTGTCTGGTAACTTTCAAGCTTTGATTCCTGGTTTTAGTTTCTCAGGCAAGAGAGTACAGGTACCAGGAAACCACTGGAGTATGCATATACGGGTCTGTCTATGGATCAAAAAATGATGCTTGCTTCAATTTTGTGCATGTTGGATCACAGTTTAGATTTCGACATTGTTCCCAGCACAATTGACACACAAAAGATTCATTGACGCTTGAGATCCAAAAAGTATAAAAGAGTAACAGGTTTCAGACTTTTTTGGATCTCTTGATTGATGTTTCCTAATAGTTGTATATCATATCTTTTGTTACTCACAAAGAACATCAAGAAATAAGCATCGAATCACATCGACTCTTTCCTATAACAAAAAAGCTTCCCTTTTCAATCCAAACATTTCAATTTATACA |
| POL4 | AATAACAAGTAAGTGCAATGCTAAAAAGAATTCTGGTTCTTGATTTTGATGGGACCATCACCACGAAAGACACTACTAGTCTGATTGGCGAGTCGGTGTACAGGCTAAAGGACGTGAAGGTTCCTTGGAATCACTATACCAAGGTTTATGAGGCGCACCATGTTCCAAAACCTAGAAGTCTGGGTACTAATCCATGGATTACGGTTTGTAACTACGAACGCGATGCCAAAAGCTGTGAGCTGGCGTCCATTAACGAGCTAGAAGCGCAAGATCATTTCAAAGACGTGGTCATCCAAGATCTTCTGCAAGTGGTGAAACCGCAGGTTCAAGTGCGGCCTGGGTTGCGTGAGATGATGGGCCAATTTCACGAGACGTACATTGTGTCGTTGAATTGGTCAAAAGATCTGATTCACGATCTGACCGGTGTGTGTAAGGGCAATATCTTCTGCAATGATTTGATATCACGTGACAATGTTACTTACAACGGACACTTTACAAAGCAAGTGGTTACTGGACTAGACAAGTACAATCTCTTGAAGGATAAGATTCTTGATCCAAAGAGTGAGGTGACGTATATCGGTGACAGTTTTGGTGATCTTCCCTGTATACTAGCAGAGGGAGTTAAAGGGTATATTATTGGTGATTCTTTGAAGCATTTACAACTAGATGTTCCCACTATTCAAGGGTTCGATGAGGTACA |
| HSP26 | CGCGCATGACAAGGGTGCATGTCCCCTTGGCACAGGGTTGACTCTTTTTGAGACACAACACCTGTGTGCCTGGCTTGGCCTGGCCTAGCCTGGCCTGGCCTAGCACCATGGGGCCGCTTCTTAGGGGAGCCATAATTCTCGGGCATAGAAAACCCCGTAGTGACGGTTGCTAGAAATTTCTGGACTGGCATCGAAAAAGTATTACCCGGCTGAATTATAGAGGAAAGTTAGCGTTATTTTCATTGATTCGCTTTTCTTTTTGTAGTTGCCGACAGTGCGGAACAGCGACAGTTTAATGAATGTGATGAGCTAAGGCAAAGGGTAATTAGAATTAGACACTAGAACGAAGGGGAAATAGGGAGGAAGATGTTACGAGAGAGTTCTTTGCATGTAGTTGTAAGGTGATTAGGGATTGTGTAATAACTTTAATCCATATAGTAGAAAGTTCTAATGGTTTCTAAGGGGTAGTTGCTGTGGTATACAGTGCGCCAGTATTATTGGTTTGGCAACAGTGATGGATTTGGTGGACCTGAAACGTATAAATTAGACACGAGTCTGGCAAGTTGATGAGAGATGGGAAGGTTGTTTCTTGTATTAATTGTATTCAGTGTTATTTCTATAGCCCCAATCACCAGAGATATTTGATTTCATAAGAGACTATATCTAAGAGATTAACAACAACTAAGAACAAAAGCGATTACGAG |
| SSA3 | CTTGCCACTATCAAGTGAGCGTCCGCGCGCTTTCCAGAGTGGCCTAGAATGTGCCGGCACCCTCTGGTCTGGAGCGAGGGCCCTTAATTTTTATTGGATACCCGGAGAATTAAGGGAAAAGAAGAAGCTTCTCGATGGGCCCACACCAGCAAATGCCAGTAGTAGTGGCCGCAGTGTAGTAGTAGTAGGACGCACCGTATGGTAGTGCCATTTGGTAGCGGTGCTACTAGAACACACTAGCCAGGAACTAGCCAAAGGTTGGTGCCCACCGGTTTTCGTATGTCTTTTGTCACTAAATCGGGTAATTGTGCTACTGTTTGATTTTGATTTCGTAATATGGCAATAGCAAGCTGAAAGTGTAAGTAGCGCAGAAGACTGTGGAATGTCATAGAATAAAGGGAGATTCGTTTGGTCTGACACTGGAAGGGCATATTGGACAGTGGAACACTGGGGGGTGAGAAAAGGTATATAAGGGGAGCTGTAGAGAAGAGTATTGGGAATTGAGGATGGTTGAGAAGTTCAATTGCAATAAAGTATTTGTTGTAATAAAGCTTATGTGTTAGCTATACAAAAGCATCAAGCCACTAACGAAAACTATTAAGTTAAACAAAGTAAACTGTCGCCAAGACTCTTTATTTTAAGTGAGTGAATTTTTACAGAGCTAAAAAAGAAGAAGAATT |
| ZWF | TAAAAAAAAAGGGTGGGATTGCATCATATATCACATTTAACGACAATGGTGGGGTGGCACCAACTACTTTGCACTCTTTAACTATGGTTTGTCGCTTGATAAGGTAAGGCTCCGCATGTAGTTTAATGAGGAATGTGGGCGGCATCGGAATGCAAAACTGTAGCTACCATTTGGTTGTTAAAATGCTTGCCAACAAATCATTCAAAAAAAAAAAAAAAAAAAAAAAAAAAAATTAGATAGCTCGGTTCTCCATATGTTGTGTATTATAAATCAGTTTTGCTTGGAACGTTATGACATAGAAAAATACAAATTATAAAAAGAGGGTTCTACTAAAGCGTAAACGTAATATAAAAAGTAGAATTGTTCATTCGTTTTTTGAGTTAGTGGAGATTGATAAGGTTTTATTGCAGCAGCTTAGTGTGTTCTATCCAATTGAACTTCTTACAGAGCATTGTTTTCTTTCGCTTTGTACTGTTTTTAGTCACGCACCCGCATACTCCTTATACTCATTGGATACTCATTTGCTTACAGAAAGTGTTAAGAATACCCTTAATCGAATAGTAAAGAAGAACAAGCAAAAGCTTCTGTCGCTGTTCTGTTTCCTGTTACTCCAAGTTTTTATCTGTGTCGTTTCAATTTCACGTCGATATAATTGCATATCATTTCTAAATCGTAACATAATACTGAACATTTGAACAAG |
| SCL1 | CAGGCTTGCGCTTCTCAAACACGCCGTGGCCGATAAACTGAAATATCCAGCTCACAACAAACAAAATCCAGGCTGTTTTAGCAGTAATTTCCACGGTCCCATCGTTCAAAACTTTGTTCATCCCAACCAATATGGCAGATGCCAAAACACCAGTAGGAACTTTCAATGCTATGTAGAAAATTCCAAACGCCGCTGCAAGTATCTGGCTCAGCTTGTACCCACCATAAACCTCCACGTCGTTCAAAATTCGCATGCTTGTGAACAAAATCGTGGGCACAAAGATACTGTGAATGAACACGTTCCCCGACGTATTGTGATACGCCTTATAAAAGGCCAACTGCGCTTCAAGGTCAAAAAGATTAGAATTAGAGCTCATTGCCAGATTATAGTTACACCTCTAATCTACCACCCAAATACAAAAACTCTTGGCCATGTACCTACTACTGTTCATGTTCTCCCCTTAACAGTCATCTAGTTACTCAGTTCTCCATCTTTTTTTTTTTTTTTATTTTTCGTTACTATTCCTCTCTTTATATATTTCATGGGAAAAACCAAAAGTTTAGAGAGGAGTAAAATAAAGGATTTCGAATTCCAATTGAAAACCAATAGACAAATAGCCTATGAATTTTCTTGGTGGCAAAACAGAATAGAAGAGAGTAGAGATAACAGCCACCGAGTGAGCGTTATCAAGTGTGAACAGA |
| ALD2 | CTCTAAAACCCATGTCGCATTAGCCGTGTGCTAAGCGAGTCCTCCGCCGCTTAGCGCAACCATCGCCGCTTAGCAACGAGTCGCTTATCTCTCTGTGTCTGCTGCGCTCGGTGTTGAGTCAAGCCCGCTTCGTATGGGAACGGAACGGCATATCTTTCCTTATCCGGACATTCTATGCGCACCTTAGATCTATGTTACCCGGTGGACGGACGAAAAGAGGGAAAAAAAAAAAAGAGAGAGAGTTGTGTGGGTGTATCTCTTTGTCTTAGGCGGTGGGGTCGGGATGGACAAGGAATTCAGTTCCCGAAATAACTACCGAAATGCGTTCGGGTTCTTGCTGCGTTTTGCTGTGCTATGCTGTGCTTTGCTGCGCTGTGCGGTCAGTGGAAACTTCCTGGGTCCGCGGATAGTTAAATAACGGGGGAGGCGGGGGGAGAGGGTTGTGTCACGTGATCTTGTATCTTGAAAACCTAGTAGGAGTGGTACTCTGCTTTTCTTCAGCCTTTATTGGCCCAGCCCAGTGGGTTGGGAGCCTTGCGGCTCCCCTTCTCTGGTATTAACCCTTCTACCTGTGACTATCAATGACTACCTCTGGGTGCTGTTTAGATAATGAATCTGATAATGAAAAAAAAAAAAAAGGAAGCATACAAGCTTGTATAAAAGATATATAGAAATAAAGGGAAGAAGAACCCGATTCCCTTGGGAATTATAAGCTGTACGGTGGGTCAGGGTCCATCAGCCGTATACAAAAGTAGAAAT |
| PST | CATTTCAGAGTTCTTTTGTCTTTTTTAATCTTCAATCTTCGGCTTTTTGTCATCTTATTAACATACAATTCAAGTACATTTACATTTACAATCACAATCACAATCAATCATCAGTCATTGGATCTTTTTCTCTTCCGAAAAGATTCGTTTAAACAAATTAAGATAATGCATATAACAAAGTACATCGTTAAAGAACATTTAACAGTATAGTTCATCATATAGTAACCACAACATTACATGGATATTCTGTCAAAAACAGAGTAGTTGGTTTCAACCCTACTTAATTGTTTTTTTTTTTTTTTTTTTTTTTTTTTTTGATATAGCTGAAGAGTAAAAAAGTCTTGACAATGAAAAAACAAAAATCCAGAAGGGAAAAATAGTGACAGGCGACAAATGACAGATGACAGAAAGCAGAGATTTTCGGTTGAAAACAAAAACAGTATGGAACAGTGACTGACGCAAAACCACAAAGATCCAAAACGTGCCAAAATACCCTTTTGTGTTGAGCAATTTTCAAATATATAAATATGCGACACTTGATGAATACAATAGAAGCTTCCTATACATTATTATAATTATCTTTGTTATTGAGACAGAGTATTTTTGATATTGTTTTCAGTTCAGAATACTGTAAGTACAGGGCTTCTAATATTCTAGGGGAGTATATTATAAGTGGAGTGACCTGTTCATATTAACAGAT |
| GLK1A | CCTTCACAAAAGAAAAGTATTTTGTTTCTACACGTATTTCTGGTTATGGAAGGTAACGTTATAGTTTTAGTAGAAGTCAGCTCGGTGGTGGTAGTGGTGGTACCTTATTGATGGACACACGAATGCCCGTAACACTATTGCCTACACACAGAAGCTGACTAGCTAGTAGACCTGACTGAAGTGCCGTTTTCTAGCATATTTTTCTTTCTGGGCTTAGTGTCCTGACGCCCCAGCGTCAGTTCCTTTAATTTGCCAGTGTCACAGACTGGTCCTTAGATGGACTACGAGAGCTTTACCCACACATAACGTGCTTCACCGTTACTATGTATGCTACGTATATACGACAATATTCCTCCTGTCTTTTCTTTGCGTTCCTCTTTCACTTAATATGGGAACCATTTCCTATTCTCACCACACATTCGCATTCGGATTCTTTTTGCTGACGATCTCCTGTTATTATTGCTTTACTCAGAAAAAACGACACATTCCCTTTATTGAAATAAAATGAAAGACGGAGTCTATATAAAGAGAAGAGGAGATGAGAACAGACCCTGTTCAGGAACAGATTATGAAAACGATTATGGTATTCTTTACTCTCGAACTTTAAACTCGAGTGAGAATCCGCTTAAAAATCAAGTATACCAGATACCGGAAACTAGTAACATAAAACTTAAAT |
| COX20 | GAAAATTGTCATAAACGTTCCTCTGCTGGTTGCTTGCCATGCTTACGGCTTCTCTTCTTTTTACCGTCTCTATCTATCTATTCTTTCCGTCCAAAAAAAACTGTCTTTCTCACACACACTCAATTTCCCACGCCTTGGACGTTTCGAATGTCTGACAGAACTGCTGCTCAACAGGGTCCCACCTAAGCGTTCAAACTTTTCGTCTCAATCTCTATATAAACTCCAGTTGGTTGGTTTTCTTTTTGGTATCCAAAACCGATATTTAATGTCCAAGAACGGACAGAGACAATACATGAAAGAGTAACAGAAACAAGAATGCTGAAAGCCGAGCGAACGGATTATGCAAGAAAAGGGAGGGGATAAAAGAGTACTGAGATCTGCACTGTCACTGGCACAGGCCACTTCTGTATCAACTCCTCTTGTCAATATAACCCCGGCCGTTACAGTAGAAAAACCACCTGCTGGAGCCGGTGGACACACACTGGTACACTCCAGCCAACTCTTTCCAGCCAAGACTTACTCTGCTTTTTCCCAATTTTTCATATATCAGTCTACTTACATACAGAAAGTAAAGCATTAAATATATAATAAATAGAGTTGAGAAGAGCAAGCGTGAACGTTAACGTTTGGAAAGAAGCCAGTTGGAATGGGCTTTTGAATGTTGTAAATACACAAGAAACCCTGGAGATTGGTGTACATA |
| SOD1 | CAGAATATTTTTTTTTTTTTTTGTTTTTTGTTTTGTTTTTCGTTTCCTTTCCTTTCCTTTCCCGTGGCGCTCGGCATTGCTCCCTTCTCCAAGCCCAGCCATGTATAACTATTACCAATCACACACCTGCGTATTACCAAATACTCATTAATGCTTGCGCTTGTGCTTGAGCTTACCCTTACTTCGTCAACCTTGGATAGCACACGGTACAAGGCGTAGCGCAACCCCTGTGGAGAAAGCACACCCACACACACACACACACCCTATGTACCCTATATATTCCTCTGTAATCCACCCCTAACACGCTAGTCCCTGGCCGCTACGTTACTACGACTCAGCACGCTTACTCAGCTAGTCAGCTACTTACTAGTCAGCTAGCTAGGCTCTACCTGCATCACCCATTTAGCTATTCCCGCTCTGTCTCTTTGTAAGTCATTTTCAGCTCCTTTTCCGTTTGCTTCTTCAATTGCCTTCCTCCATTCTTCGGCTCCCCTTCGATTTCTAATCGGGGACTGGGCAAAAAAAATTGGAAAAAGTATATTTAAAGGTTATAAATTTGCTCTTCTTTCCTCCACACCCTGAGCACTAGGCAGTACATTTGCACGTGAAAAAGGGTAAATTAGCTCAATTCAATCAATCCAAAGGTTAGTTACACTAGACCCACCGCAGGAAGGAAACATAACACATACAATTAATTAATC |
| GPD1 | AAAGGGACAATCGGAGAATAGAAATCGGCATGCCCACTAAGCACGCAACGTCCTCAGGTTGTCCCTCAAAACCGAAAGGCCAAACAGCCCAAAATAAGGCCAAAAAAGGAATGAATTAAAAGAAAAGAGGACAAAATTCCCGTTTCCTTGGGTGTAGCACCGCATGACGGTGAACCTACCCCCAACCAAAACGAAAGAAGTAACAGAAATTGATGGTACAACAAGGCTGATATTGACCAAATTTCCCAGAAACACCCGTACCAGATGTTCCGCTCGGTTCTGGGAAAAATACTAGTCCCTCACCCAGTCCCTCCCCTAGTCCGATTCTCTTCTCCTTACAATTTGCCTCCTGCCCTTTGCCCCTTTGTATATACAGAAAGAAAAAAAATAATAATAATGAGGTATTACTATGCTAAAACTGAATTGTAATTAACTATGTGCAGTGGCCCTTTTTACGTTATGTATCATCATCGTCAGCATCATCATTCGCGCGTTACACTGTTTGTTGGTGCTGTTTCAGTTGGCTCTCTTGGTTTGTCTATCTGTTTCTTCTTTTTCTTGTCTATTTCTTTTTCTCCGAAACGTAGTATATGTACGTATTTGGTGTTGTATTACATAGTTGAGCCTTAGCCAGTAAGAGTTTCGGAAGGGTACGGAAACCAAAAGTATATAAAGAGAGTGGAGGAATCAGTTTTCAGAT |
| GLK1B | TTGCTAGCCGTTTTTCCAAGGTTGTTTTTTTTTTTATTTTTTTTTTTTCCATATTGTCATTTCGTCGATTTTTCGTCTTCAAATTTTTTCCAGAGTTTCCAGTATCAGCTTCCAGCTTCCACAAAGTTTGGAAATAGCAGTTGCCAGCCGTCTGTTGCCAGTTGCCAGTTGCTAGTTGCCGGCATAGCAAGTAAGAAACTCACACTTTTTTTTTTTTTTTAGTTGTATGGTATTGTACTAGTTTTCTGAAGGATGGAATGATATACGTGGAATGAGTGGTGGTGGTGGTAGGTGGGGTCTTATGGCAACTATGTTTGTTTGTTTAGAAGAAGGTCGAGTTTGGTTTGAACTTGAGATTTTTGGTGTTGGATGGAATATGGAATGGAAATGGAAATGGGAAATAAATATGAAATTATAGTTGGGAGCGAAATTTACAAATTATATATAAAGAACGTTTCTTGGATCTGTGATCTGGGGATTCGTGTTTGAAATGCTTAAATTTTTCTTGACGTCTATTATATTGTATAAATAGTTGTAGCTTGACCTTTTATTTAATTCATCTTCTTGTATTGAAAAATCAAGGAACAACTAGTTAAGCTTAAGCCAGCTAAAGTAGTAGAGTTCTTAATCGGCAAGTGATATCAAAAGCACTTAACACTCGGTTTACGCCTCCAATTATTACAAACACACACACTTACAAAA |
| HSP60 | CTTAGCTAGTGTACAAACATGGAGAAGATACAGAGAAACGACCTGACTAAAACAGAGGTTTCCCCGCTGAAAAGAATTTTCTAGAAAGGGAGGCACCAGTTTGGGCATGAATATACTGCTAATAGATAGGGATACATACATGTACTCACTCACTGTTGTTTCCATTGGAAATGCGAGTTCGGCAAAATCACGGTTAAATATGAAAAGATGGACGAAACCGAAAAACCGCGTGGAGATCCATCTCCGCCCCTCTTCCGCTTCGAAACTCACAGAACACTCAACTGACCCTTAATTACTGCTGTTTCTCAATTTCTCAATCTTGCAGCTTTCCCAGCTATTAGTTTAACTTTTTTTTTTTTTTTTCTTGTTCTGTCAATTTTGTTTTAGCATGACGGATTTTTTTTTTCTGTAATTTTCTTTTCCGTTTTTTGGCTTTTCATCGAAAAATTTCAGAGGGTTTAAAGTATATAAGAAAAGAGGATTAAGAATCTGGGAAAATTGGAAAAAAGAGATGGGAACTTTAGCGCCACTTGATTCGGATAAACAGAGTCAGCAATCAGCTATCGGTCGTTCTGTCGTCTTATAAACTAGGGAGCCGGGTTAAAAATATTGTTTCAAAAGGGGACACGTCAGGATTTTGATTGGTTAGTTTATTTTGAAGGATCAGAAACCAAGTGGATTTAAATTAAATTAAATTAAATTAATATAATACAATACAATACA |
| TDH3 | CACTATATCAGGCCTCCACTATTCCAGGCTTCCACTATCCCACCAGTTTTTCCGACCACTTATTCCGATCCGGCAGGGTTCATCCTCTCACCTCCCCCCGCTGCGCACCCGTCTAACCACCCTCGGCATGACGAATTCACTCGAGAGGCCCTCATCCACACAACATATGGCTAAAGTGGTGAAACTTGGGCCATGACAAAGGCCATGGAAAATGGTAGTACCATGGTACTACCAGTGGTACTACCACTAGTGGTGGCATTAGTGGTACCAGAACCACCTGTTGATTGATGGCTGGGACTCCTGTATTTTGGTTAGGGCCTGTCTGTTGGGGCCTGTCAAACAACCAACAACACCCTTCCATATTCTGTTCTATCCAGCTAGTTAGTTAGCTAGCTAGCTAGCTGTCTTTCTCTCTCGTATGACATGGCCTAAGGCCATGTCATGTCACACATACACATACACACCGGTTTACCGTTTCAGTAAATATCCAACATGCGATCATGCCAATATTCTGCCAATATTCTGCCAAATGTTCTTTCTGCCATTCTGCCATATATAAAGACCACATTTGATATCCAATTTCCCAATTCCAAATGATTTCGAATACATTAGTCTATAATAATTATATCTCTTCCTTGTTCCTCCCAAACCAAGTCTTTTAGATTTAACAACAGTACACACACACTTTTACACATTCACA |
| PGK | TTACCCTCACTCTTTCACATTACCCTCCCCACCATATCTTCCCCCCCCAGCTTCCTGTCTTCCACTATCCCTGCAACCACCACCACCACACAACAAACAGCCCCAAACAACCCCCAACAGCCCGGAAAAATCCACGCCTCCTCCCGAACACACGAAGCCCGCCCCGGCTTCCACTCCAACACTGCCAACACTGGAACCCCGCACCACGTAACCACCCACTTTAGTGGCTGCCCGCCCCTCCTGCGCTTCCCTTGCGCTTCCCTTGGGACCACACTCCGGTGGCATGGCCCTCGCTTGCTACATGCTACCTTTTTTTTGCCCTTTTCTGGCCTTTTCTGGCTTTTCTGGCCTCACCACAGTGTAGTGGTGTACCACAGTGTAATGGTGTAACATTGTCGAATGGTATTGTAGCCTGTTGTAGCCTGTTGTAGCCTGTTGTAGCCTGGTGTAATGTCTGGCATAGTCTGGTGTCTGGTGTCTGGTGTCTTTTGGAAACTGAACAGTGGAAAGTGGAAGTGAAAAATTGTATAAATATAGGTGTCCCATTCGTGGTTTGGTGGATGTGTCCTTGACATTGCAGGTTTCTCTCTGTTTCATTGTGTTAAGACTTGTTAAGACTTGATAAGTATTTCCCTAGTAATACCCCACCAACCATCGGTTGATACATTTTAATACACACTACCTATATAAAGATACAAAA |
| HTB2 | ATGCCCAAACCCCGTGCCATGCTATGACAATTCATACCGCTGCACTCTAGAGACGCGTCGAATTCACCAAGTAGAACGCGAAAATACGCTCTTTAGATGTGAACGTTCTGAGACTTTCGCATTGCTCTATCCTACGCATGCGAATCTCGCAGTCCTCCCTTAGATGGCTTCTTTCTCAAGAAAAATGAAACTTTTTGATCGTTCCGTTAGCTTCACCTGGACGTAACTTTTGTAGTGAATCTTTCATCACGCATATTGTAGTGGGGTTTTCTCAAGAATTTGAAAATCAAAAAAAAAAAACTAATCTAAGTGAACTTAGTGGCACGCGTTCCGTGCCAGATTGGCTCCTTTATTTCTACAGCAAGTTCTGAGGATAAGCACCCTAAATGTAAGAAACTAGAGATATGATATTACAATTTGGCGCTAAACTTGCTAGAGATGATCTTATCCTTAGTGAGAAGATCAAAAAATAAAGATTATTTCGTGTTGTTTTTTGTATATAAGAACCTCATTTTGTCGTGTTTGGTTTGGGCTGTTTAAATGGACTATTGGGGAGATAGGTTTTTGGCTGTGTATATAACATAAGTCTTTTTTAGTTTTTAGAGAAGATCTTTTTGTTTGTAATAGTTGTTTATTATATTAACTTAGTTTTAGTTAAAGGAAATCAACCAAACCAAACTAAATCACATATACATACACA |
| HTB1 | TATTATGTTGTGTTTATGTTATGTTGTATTACTTTCTTGATGAATAGTATAACTAGAAACACCACTAACTGACTAATGACAATCGGAACCCAAATACTCCACCATATCGCCTAGTGTGCTCCTTATATACGTGTAATTTTTCACATAGACTTCCCCCATCCACCGCATGCCAAAAGCAACGTGACGCCACTGTACCTCCACTGTACCACCACTATGCCTTCACTAGGCCTCCACTATGCGTCCACTATGCCTCCCTTTTCACCCCTGTTCTCCTAGCTTCACCGCTCTCGTGAAACTCCTGGAACGCTCTCATCCAGTCTCTGCTCCTCTCGAGAAATTAGTTTTTCAAGTTTCCAGCGATTTGCTCTTTACTATGGACTCCCGCGCTTCTTTCCGGATTGGGAAGGCGACTTTTTCGCGTGACGAGTGTTTCGCGGGAAGACGTCTCCATTTTCGGGATTTAGTATGGGAGAGGCCTCGGTTGCGCTGTCTTTTCCAGCGAAACTGCCGGCACGACTCGTCTCGCCATACTTAACGCGTATACAAAACGTTTATATATAAAGGCTAAGGCTAAAGGCTAATATGGTTTGTAAAGGATGCCCTGACCATTTGATGCTATTATATTGTTTTGCCACTGAATAGGTCAGTCAGTCAGTAAGTAACTACTACGCCCCCCAGATAACCAAATAATACAGAAAAA |
| HHF1 | GTAAGTAATGGAGGATTGATATGTTCTTGTTCTGTATTGCTGCCTTTATATATGGTATTGTACAAGGTATTGGAGAAATAAAGAGTGGATGCAATGGTACCAGAGGTGCCAGAGGTGCCAGAACGGTGCCAGAAAGATGCCAGAACGGTGCCAGAACGGTACCAGAGGTTCGGGGGTCCCAGAGGGTCCCAGATGGTCGTGGTTAGCCCTGTGAGGCCTTGTCAGGCCTTGTGTAGCTGGTTTATAGTGCGGTTGCCCAGAGAAACGGGGTTACCCGGGGGCAATTCTTCCATGCTGCCACCTTCCCATGCGAACCCTCTGGCACGACGCGAAACGCGAAAAAAACAAAGCCACGCGTCGTGCCAGGGCGTTCCCCCGTGAAACTTACAGCACGACAGCACGACAGCCTGCTTCTCCATAGTGTAGCGACGGCCGGTACCACGTCCATTCCAGCTGGTTCCATGGGCCTGAACCGAGCGGTTTTCCCGGGCCCTGTGGCGTGCGAAACTGGTGGCATGGTGGAGGAAATTATATATTGGATATTCCTTGGATATTCCACTATCTTTCCCTGTGTCATGCCATATATAAAGACAAGAGTGTTGCTTGAGAGTTGATACTAGACACTTGATTCTACTTAGAACATGTTATTAAAGTATAATACCCCCCACAACAAGCAATAAACAGCAATCAATAAATAAAC |
| HHF2 | CTGGCAGGTGGTTGGACGGTCATTTACCGTGCTGTGCGGTTCACACGCGCTGGACCAGAAGTTCCTCGCGCGTGCTCTGGGCGATCGTCGAAAAAAAAACAAGCCGCCTTTCTTTTTCCCTTCCAGGGGGCTCGCGCGCCCAATCTTTTCCAATCTCTGACCGCAAATGGCCATAGTATTCGCACCCGACGCCATGCTGTGCAAAGCGAGCCTGCGTTTCTGGCCGGAAGCACCGTGCTGTGCACTTCGCACCTGTGCCGCGAACCGCGCGGCACGCCTGTTCCCTCGTTCCCCAGCTGGCCTGGCAGAAAAAAAAACAAAAACGTTGGGGTTGGGTATGCGGTGCACGGGTGTGCACCGGGTAACGTCGTGCCAGGGGGTTCCCTCTTTTTCCCAGATAAATAGTGAAACGCTTGGCACGCCTTTCGGCATGGCATGGGCAATGGGCACTGCGAAACAAACGCAACAGAAACCACCTGCAAACACGACCGTTTGCTGCCGGAATACGCCCACCCCCAACAGGCGTGCTTTTCGCCGTATCTCTTGTCTCTTGTAAATACAAGATACAGAAAAAATATTTTTTCGTATATAAAGGGCCCTCCTGGCCTGACCTTGGGATCTTCCAACTGCCAAATATACTAATATACTATACTATACTATACTATACAATCCCCAAGCAACAAACAATCAATAAACAAAC |
| TEF3 | AACACCGATGAAGCAAAGAAGTAACAGCAGGAAAGAAAAACAAACACAACAAAAAAAAACAAGCAGCATAGCATCAACAGAAATTTCTAAAGAGAACCAAATTCACCCCAGAAACAACCGCACAAATACGACATCCATCCACCTTTCTTTTATCTTCTTTTTCTGATCTGATAATTAGTTTCATATACAATACGTAGAAACAGGCGCACAGCACCCAGACCTGGCTTCTGCCCCAGTGTATAAGCAATGTAGCATAATTGGAAAAAAAAACGAAAAATACCGAAAATAAGTGGGAAGCTGGGCCACAGGAGTGGGGCGGGATGCGACTGGTTCTGAGCGGGACCGGGTAATAAGGTTGAAAAACTTTGAATTGATGGAATAAGTAACTTCTTTCTTTTCGCTGGCGGGAGAAGGAAAAAAAAAAATTTTTTTTCCTTCTGTTTAGTACTGGGACATTGAGAAGGCGTGTCAATTTTGAATAATTAGAGTGGTCAAAAAAATTTTTTTTGCTTGGGATACCCTTTTTCGATAATGTAAATTTTTTTTGAATATAAAAGGAGATTGAAAAATTTTTTCTAGCAGAAATGTTTTCAAGTTTTAATTGCAAGTTTCGTTTGAGTATTCAGTTGTATTTTAGTTGATTTGTAGTTTATTTACTAGTATTCTCATAGTTCTAACTCCAAGAGAAGTAACATTAAAG |
| NC1 | CCACGCAGTGTGAATGGACTTTCGAGAGTGTTGTTAAATCCTGCGAGATTTCTGGTGTTTGTGGTAAAAGCATGTAAAGTTGTATACTGAATTTGTACGCAGTTTCTAAGGCATTTCTTCTTGTGCACAAGAGCAAAAGAAAAAAAGCAAAATTTATGAAATGACGAAAAACTACAAAAAATAAAAAATAAAAAAAAATAAGACGTATTATTTTTGGTGGACACGCAGACCACGGCGTGCAAAGCAAGTTGTTTTTGTTTTTTCTAATTGGGCAGTTGGGAAACGGTGAAAACTGTGAAGAAAAAAAACAACATTTTTCTTTCTTTATGCAATACCAATTTGGAAATCTCGCGGCCGTGCGTGTTTTCTAGGGCGGGTGGGATTTTTTGACAAATTTAGAAAAGATGCCACCAGAAATAACTTTTTTTAGATAGTGTTTTTTTTTCTAGTTGTTGTTGCTAGAGATCTTTTTCAGAAACGGAACGAAACGAAAAAGGAGACCTGTGGGTGGTGGCGATAGATTACCAGATGGTCGAGAAATTTTTTTTTGAGTATATATACAGGGGGTACGTTTCAGAATTTTGAAAACCTATTCTTCCTTCTCCTTAACTCTTTTATTATAATTATTATATTATTCATTTTAGACTTAAAAAACCATAGAATAATATTCTTCAGTCACTCGCTTAAACACAAATCAAAA |


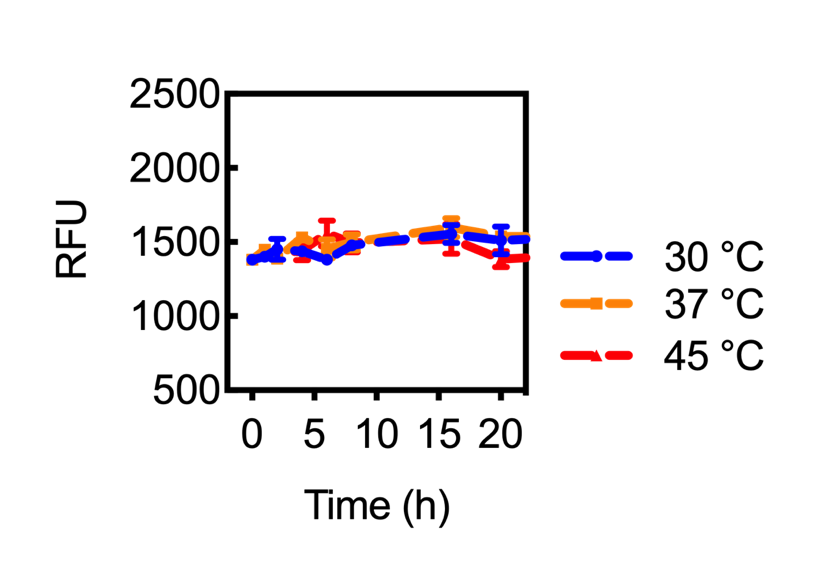


Figure S1: Thermal stability of EGFP. The cell lysate was incubated at 30, 37 and 45 °C for different times. The fluorescence signal intensity of incubated lysate was measured at 0, 1, 2, 4, 6, 8, 16, 20 hours by BioTek®Synergy™ Neo2 multi-mode microplate Reader (excitation: 488nm, emission: 511nm).


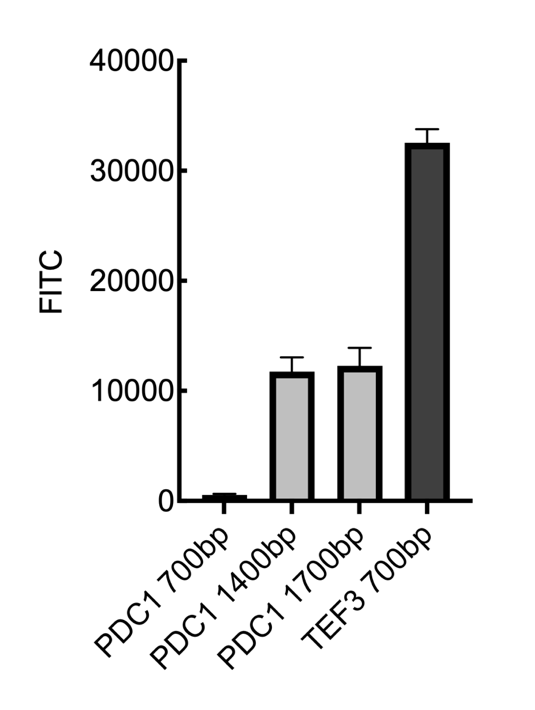


Figure S2. Truncated P*_PDC1_* and 700 bp P*_TEF3_* fluorescence signal intensity determination by flow-cytometry. After 14 hours of culture, cells were harvested by centrifugation at 5000 rpm for 1 min, then washed twice with PBS. All the samples were diluted 50 times in water before measurement. The mean fluorescence signal intensity per cell was measured on a BD Accuri^TM^ C6 plus flow cytometer.


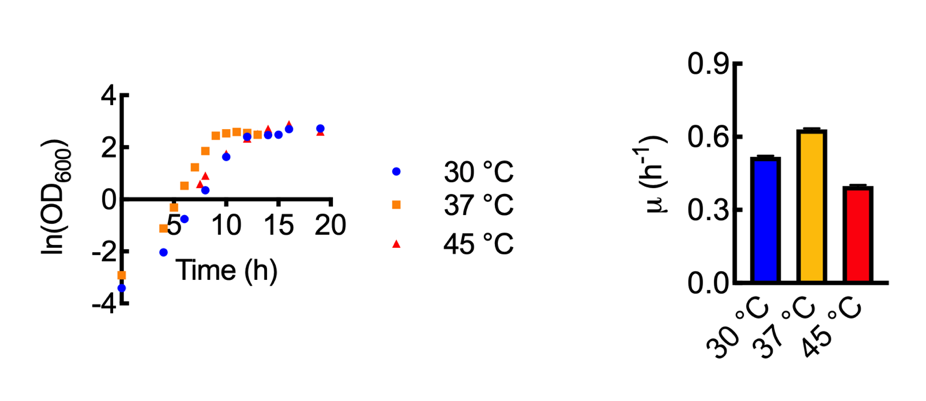


Figure S3. Growth curve (left) and growth rate (right) of *K. marxianus* CBS6556*𝚫HIS3𝚫URA3* in 2% glucose harboring a low copy number blank plasmid (pIW 578). Shake flask cultures were inoculated with an initial OD_600_ of 0.05. Triplicate cultures were grown at 30, 37, and 45 ºC.


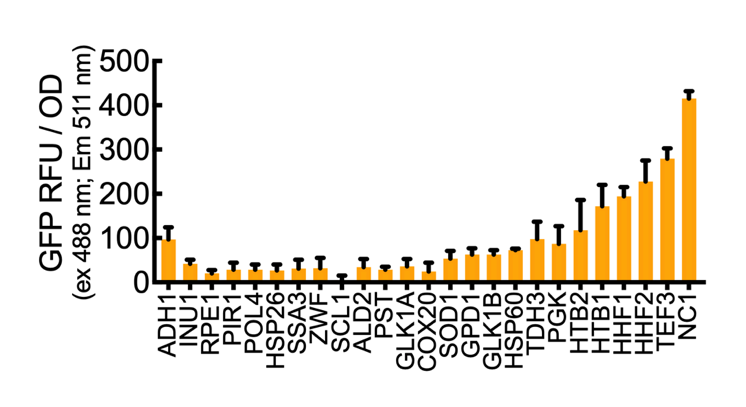


Figure S4. EGFP fluorescence per OD at 9 h and 37 °C of 25 *K. marxianus* promoters in 2% glucose. Data points and bars indicate the mean of biological triplicates. Error bars indicate the standard deviation.


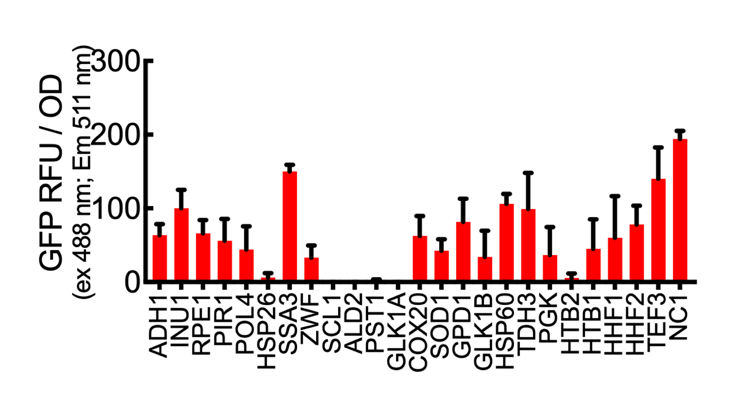


Figure S5. EGFP fluorescence per OD at 14 h and 45 °C of 25 *K. marxianus* promoters in 2% glucose. Data points and bars indicate the mean of biological triplicates. Error bars indicate the standard deviation.


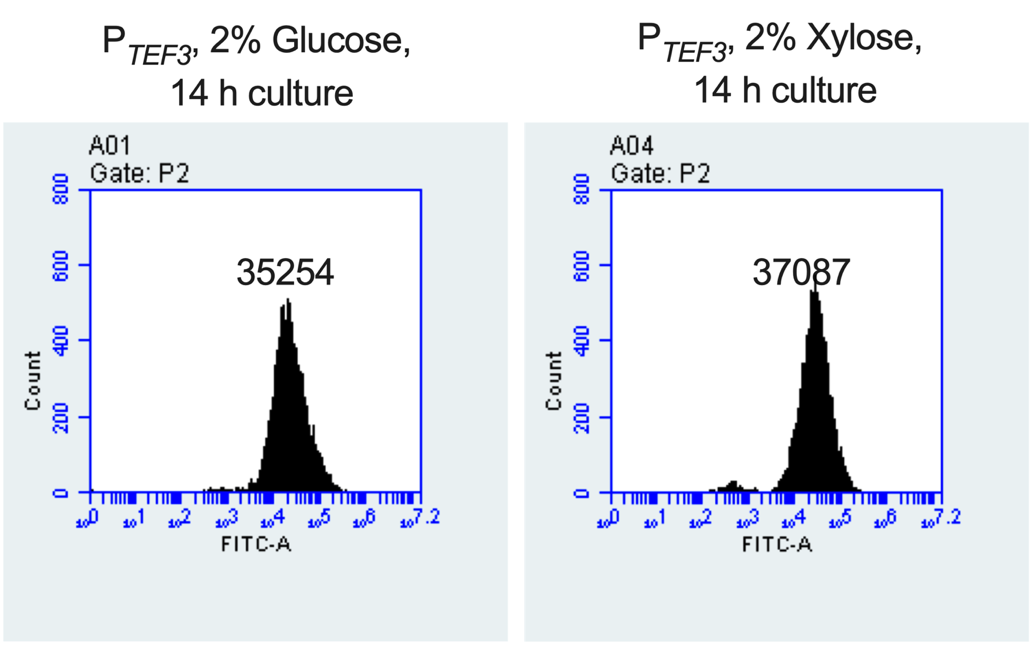


Figure S6. The flow cytometry results for *K. marxianus* CBS6556 *ΔHIS3 ΔURA3* cultured in two different carbon sources, glucose and xylose. CBS6556 *ΔHIS3 ΔURA3* with P*_TEF3_*-driven GFP expression plasmid was cultured in 25 mL defined media without histidine. After 14 h culturing at 30 °C, EGFP fluorescence signal intensity was measured by flow cytometry.


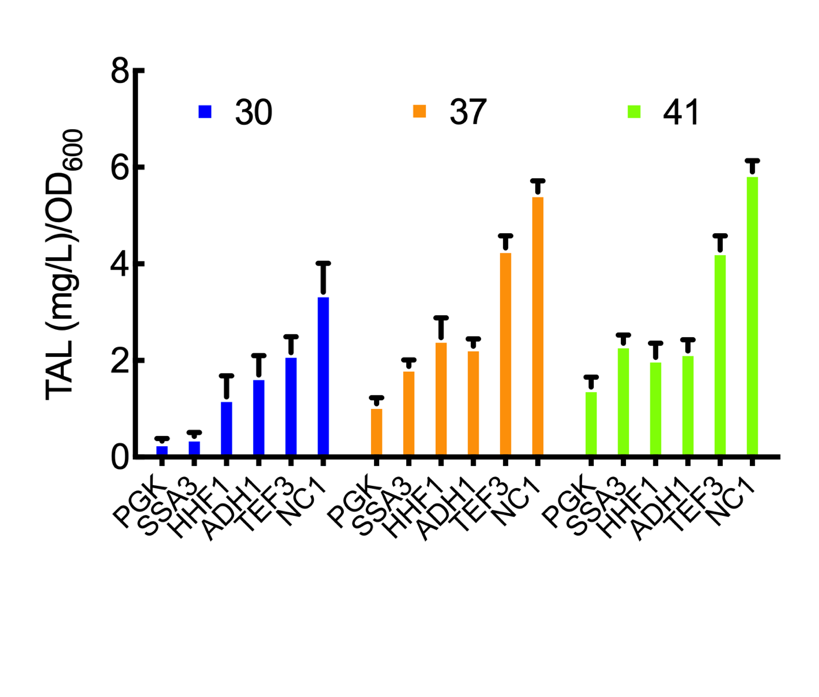


Figure S7. TAL biosynthesis in xylose medium with varying levels of 2-pyrone synthase (2-PS) expression. Specific TAL production was measured at late stationary phase at 30, 37 and 41 °C. 2-PS was overexpressed with *PGK, SSA3, HHF1, ADH1, TEF3*, and *NC1* promoters on a low copy number plasmid.


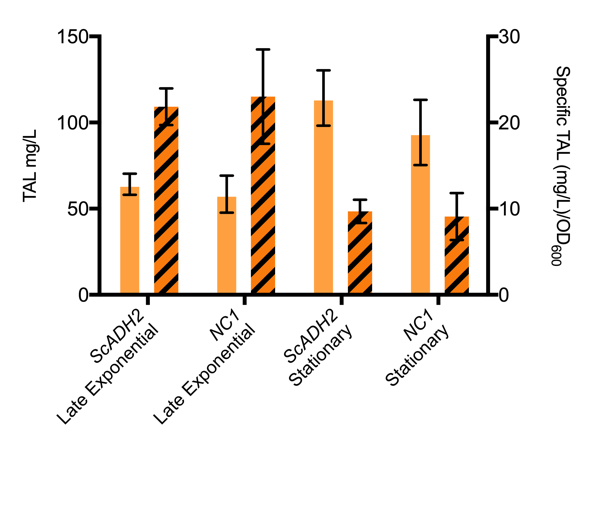


Figure S8. TAL biosynthesis in 2% SD-His medium for CBS6556 *ΔHIS3 ΔURA3* cells transformed with P_Sc_*_ADH2_*-2PS and P*_NC1_*-2PS. TAL titers (solid bars, left y-axis) and specific TAL production (slashed bars, right y-axis) were measured at 37 °C in late exponential phase and stationary phase.


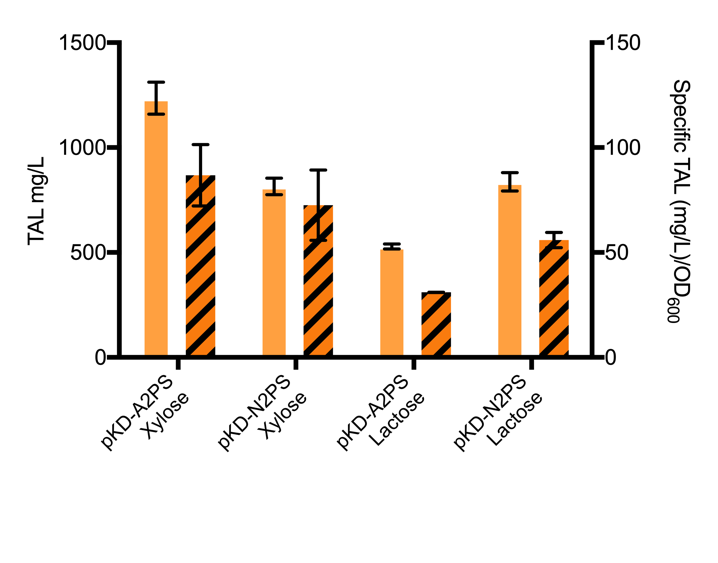


Figure S9. TAL biosynthesis in SCA medium containing either 1% xylose or 0.95% lactose as carbon source. KM1*ΔURA3* cells were transformed with pKD-A2PS (P*_ScADH2_*) and pKD-N2PS (P*_NC1_*). TAL titers (solid bars, left y-axis) and specific TAL production (slashed bars, right y-axis) were measured at 48 h, 37 °C.


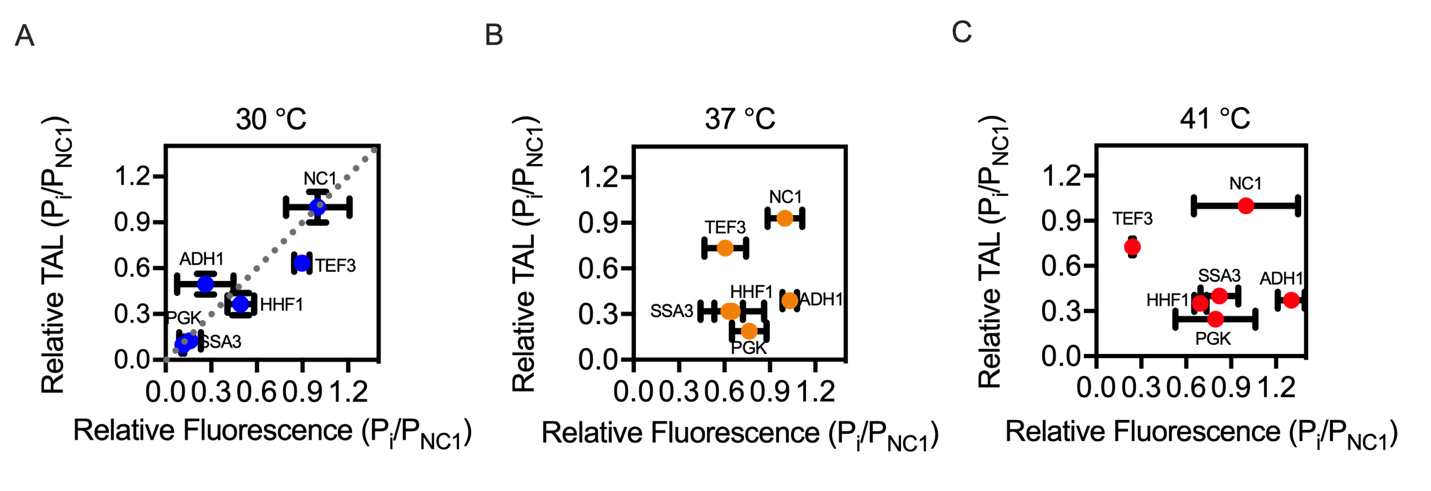


Figure S10. The correlation of relative TAL production level and relative EGFP fluorescence signal intensity. *K. marxianus* CBS6556 *HIS3 ΔURA3* was cultured in 2% xylose at different temperatures (30, 37 and 41 °C). The TAL production level was normalized by that using P*_NC1_*. The EGFP signal intensity was normalized by GFP intensity using P*_NC1_*.
